# Studies upon Fluorescent Modulation of Silver Nanoclusters Formed on Bifunctional DNA Template

**DOI:** 10.3390/ijms26104914

**Published:** 2025-05-20

**Authors:** Patrycja Filipczuk, Agnieszka Fedoruk-Wyszomirska, Joanna Nowak-Karnowska, Zuzanna Pietralik-Molińska, Ewa Banachowicz, Maciej Kozak, Anna Dembska

**Affiliations:** 1 Faculty of Chemistry, Adam Mickiewicz University, Uniwersytetu Poznańskiego 8, 61-614 Poznań, Poland; patrycja.filipczuk@amu.edu.pl; 2 Institute of Human Genetics Polish Academy of Sciences, Strzeszyńska 32, 60-479 Poznań, Poland; agnieszka.fedoruk-wyszomirska@igcz.poznan.pl; 3 Department of Biomedical Physics, Faculty of Physics and Astronomy, Adam Mickiewicz University, Uniwersytetu Poznańskiego2, 61-614 Poznań, Poland; zuzanna.pietralik@amu.edu.pl (Z.P.-M.); ewa.banachowicz@amu.edu.pl (E.B.); maciej.kozak@amu.edu.pl (M.K.); 4 SOLARIS National Synchrotron Radiation Centre, Jagiellonian University, Czerwone Maki 98, 30-392 Kraków, Poland

**Keywords:** silver nanoclusters, G-quadruplex, potassium sensing, fluorescence

## Abstract

The use of DNA as a scaffold for nanoclusters is particularly interesting due to its structural versatility and easy integration with aptamers. In their structure, aptamers often contain non-canonical forms of DNA, i.e., G-quadruplexes (GQs). Four-stranded GQs are used to construct nanomachines and biosensors for monitoring changes in the concentration of potassium ions. In the present study, we continue our work related to the synthesis of silver nanoclusters formed on a bifunctional DNA template. By attaching a cytosine-rich domain (C12) to a G-quadruplex-forming sequence—human telomeric (Tel22) or thrombin-binding aptamer (TBA)—we constructed bifunctional templates for fluorescent silver nanoclusters (C12) with the ability to detect potassium ions (GQs). The changing localization of the C12 domain from the 3′ to 5′ end of the oligonucleotide was a successful way to improve the fluorescence properties of the obtained fluorescent probes. The best performance as a probe for potassium ions was exhibited by C12Tel22-AgNCs, with an LOD of 0.68 mM in PBS. The introduction of the fluorescent cytosine analog tC leads to an LOD of 0.68 mM in PBS and 0.46 mM in Tris-acetate. Additionally, we performed AFM, TEM, DLS analysis, and cellular studies to further investigate the structural properties and behavior of the Tel22C12-AgNCs in biological contexts.

## 1. Introduction

Scientists pay special attention to metal nanoclusters, which are nanoparticles with tens of atoms and a diameter of 2 nm that emit fluorescence [1,2]. Nanoclusters with a size approaching the Fermi wavelength of electrons display discrete, size-tunable electronic transitions, and intense fluorescence emission as a result of their substantial confinement effect compared to metal nanoparticles [3]. Silver nanoclusters (AgNCs) have attracted special attention due to their facile synthesis, tunable fluorescence emission, and high photostability [4,5,6]. To prevent the aggregation of silver nanoclusters and their oxidation, a stabilizing scaffold is required (matrix) [7]. Due to the strong interactions of silver cations with bases and DNA phosphate groups, it is possible to design and manufacture DNA-based silver nanoclusters (DNA-AgNCs). In particular, Ag^+^ ions show a strong binding affinity to cytosine bases (C), forming the C-Ag^+^-C complex [8].

Especially, compared to organic quantum dots or fluorophores, DNA-templated metal NCs display better inherent features, such as strong photostability, biocompatibility, low toxicity, big Stokes shift, and tunable light emission wavelength [9,10,11].

Silver nanoclusters have gained attention across various scientific disciplines due to their unique properties and versatile applications. These nanoscale structures, comprising a defined number of silver atoms, exhibit distinctive features that distinguish them from bulk silver and other nanomaterials [12]. Understanding the synthesis methods, structural characteristics, and potential applications of silver nanoclusters is crucial for advancing our knowledge in nanoscience and technology.

The size-dependent properties of silver nanoclusters, influenced by quantum confinement effects [13], have led to considerable interest in their synthesis and exploration. Various techniques, including templated growth [14] and bottom–up approaches [15], enable precise control over the size, shape, and composition of these nanoclusters.

Silver nanoclusters demonstrate unique optical properties, such as surface plasmon resonance [16], opening up possibilities for applications in sensing, imaging, and optoelectronics. Additionally, their catalytic activity has prompted investigations into diverse applications, from environmental remediation to energy conversion [17]. The biocompatibility of silver nanoclusters further expands their potential applications in biomedical fields, including drug delivery [18], imaging, and therapeutic interventions [19].

In 2023, researchers [20] investigated silver nanoclusters (AgNCs) stabilized on a specific DNA hairpin structure. By utilizing DNA as a template, the researchers achieved precise control over the clusters’ optical and structural properties, resulting in an elongated structure with enhanced fluorescence. Additionally, the study explores the antibacterial potential of these AgNCs, suggesting that their interactions with molecular oxygen could underlie their mechanism against bacterial cells.

Another review [21] covers recent advances in the synthesis and functionalization of silver nanoclusters (AgNCs). It discusses methods to achieve atomic precision and how specific ligands can influence optical properties, enhancing applications in fields like catalysis and optoelectronics. The study also explores how AgNCs’ structural transformations under different conditions affect their stability and reactivity. It highlights AgNCs’ potential in energy conversion and fine chemical synthesis, offering insights into their catalytic mechanisms. Future directions focus on overcoming challenges for broader application in emerging technologies.

In our previous work, we obtained highly fluorescent AgNCs on a DNA template consisting of a cytosine-rich (C12) domain integrated with a guanine(G)-rich domain [22]. The main idea of such a constructed DNA template is that the C-rich domain is mainly responsible for nanocluster formation and serves as the fluorescent tag, whereas G-rich DNA can form a G-quadruplex and serves as a receptor layout for potassium ions. Our studies confirmed that the competitive formation of the G-quadruplex structure as a result of the binding potassium ions has a significant impact on the emission properties of silver nanoclusters, and such a probe can monitor minor changes in the K^+^ concentration in the extracellular conditions [23]. The presence of a G-quadruplex domain responsible for potassium or sodium binding avoids the situation observed by Francos et al. in the case of AuNCs@GSH [24]. They proved that sodium and potassium, instead of forming complexes with GSH, exhibit moderate binding constants to induce the efficient aggregation of negatively charged AuNCs@GSH via the formation of inter-cluster electrostatic linkages and aurophilic Au(I)—Au(I) interactions between the closed-shell metal centers [24]. Recently, numerous gold nanoparticle-based colorimetric aptasensors for potassium determination have been proposed [25]. Some of them have also utilized G-quadruplex-forming sequences to recognize and bind potassium ions [26].

The potassium ion plays a key role in human organisms. It occurs in human cells at a high concentration and controls, together with Na^+^, the osmotic pressure in them. It also maintains the homeostasis of the cell volume. The concentration of K^+^ is balanced by the concentration of, e.g., Ca^+^ or Cl^−^ in cells [27,28]. Therefore, any change in relation to the norm of the K^+^ concentration in the cell or blood or any defects in K^+^ channels can cause some types of disorders or diseases, such as hypertension, heart disease, seizures, or strokes [29,30]. Therefore, real-time monitoring of K^+^ changes is an important task. One should remember that sensors developed to monitor intracellular and extracellular K^+^ must exhibit high preference for K^+^ over Na^+^ [27]. For example, to probes with high selectivity of K^+^ for Na^+^ belong TAC-Red, a triazacryptant (TAC) type [31], as well as GEPIIs [32]. On the other hand, there is still a need to develop fluorescent bioimaging probes for visualizing K^+^ transfer through cell membranes [33,34,35].

The aim of the presented studies was to improve the optical properties and stability of fluorescent AgNCs formed on a bifunctional DNA template by changing the location of cytosine-rich and guanosine-rich domains. The G-rich domain is derived from two G-quadruplex-forming sequences: human telomeric (Tel22) or thrombin-binding aptamer (TBA). Therefore, we synthesized DNA-AgNCs on four templates: Tel22C12, C12Tel22, TBAC12, and C12TBA. The studied systems differ in the attachment of the C12 sequence: Tel22C12 and TBAC12 have a C-rich domain at the 3′ end, whereas C12Tel22 and C12TBA have it at the 5′ end. We used UV-Vis, fluorescence, and CD spectroscopy to monitor silver nanocluster formation and to perform spectral characterization of the obtained TBAC12-AgNCs, Tel22C12-AgNCs, C12TBA-AgNCs, and C12Tel22-AgNCs. We selected C12Tel22-AgNCs as the system with strong fluorescent and stability properties and introduced the fluorescent analog of cytosine, tC, to the C-rich domain of that system. Our goal was to introduce tC, a fluorescent analog of cytosine, into the C-rich domain, which is responsible for silver nanocluster formation, to force better fluorescence brightness of our probes. We hoped that the replacement of cytosine with its fluorescent analog 1,3-diaza-2-oxophenoxazine, tC, would allow for controlling the intensity and sensory properties of the resulting silver nanoclusters and would lead to nanoclusters emitting with higher intensity (higher quantum yield) and a maximum emission peak located >650 nm. Finally, we explore the ability of modified and unmodified C12Tel22 silver nanoclusters to serve as potassium probes *in vitro* as well as *in cellulo*. Additionally, we tested cholesterol-modified C12Tel22 silver nanoclusters to serve as a probe for the visualization of transmembrane transport of potassium cations.

## 2. Results

It is known that the sequences and lengths of the template DNA strands play significant roles in determining the sizes of the DNA-AgNCs and, thus, their optical properties; in contrast, the Ag^+^/DNA molar ratio determines the fluorescence intensity [4,36,37]. Therefore, in the first step of our study, we focused on comparing the properties of DNA-AgNCs synthesized on the oligonucleotides Tel22 and TBA possessing a C12 domain attached at the 5′ end or at 3′ end (see Section 4.1 in Materials and Methods). Silver nanoclusters were synthesized following the procedure described at our previous work [22] in two different buffers, as described in the experimental part. The formation of the nanoclusters during the reduction step was evident from the appearance of a yellow color. We used the UV-Vis spectroscopy technique to monitor silver nanocluster formation in PBS as well as Tris-acetate (TRIS) buffers (Figure S1). As expected, the most pronounced differences were noticed in their fluorescent properties.

### 2.1. Fluorescence Properties of AgNCs Templated on C12 Integrated with G-Quadruplex-Forming Sequence

**Figure 1:**
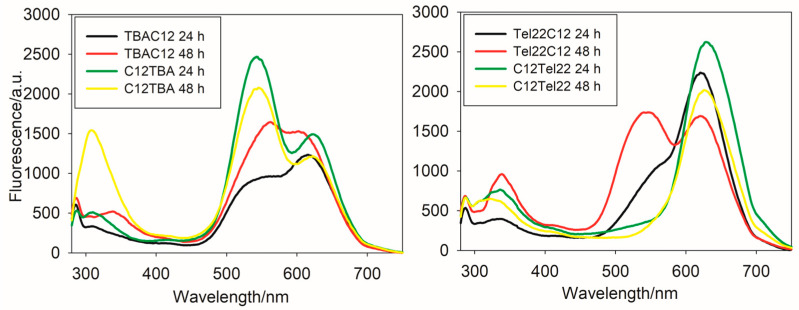
Emission spectra upon excitation at 260 nm of TBAC12-AgNCs and C12TBA-AgNCs (**left**); and Tel22C12-AgNCs and C12Tel22-AgNCs (**right**); conditions: DNA (2 µM); PBS buffer; pH = 7.4 (0.01 M); DNA:Ag^+^:BH_4_^−^ = 2:1:1. Conditions: Ex bandwidth: 10 nm; Em bandwidth: 10 nm; response, 0.2 s; sensitivity: medium; measurement range, 280–750 nm; scan speed, 1000 nm/min.

Figure 1 and Figure S2 show the emission spectra of the obtained nanoclusters in PBS buffer upon excitation at 260 nm and 570 nm, respectively, and Figure S3 shows their emission spectra in Tris-acetate (TRIS) buffer. The emission spectrum of TBAC12-AgNCs in TRIS exhibits maximum fluorescence at lower wavelengths than C12TBA-AgNCs. The emission spectrum of C12TBA-AgNCs in PBS or TRIS displays two distinct peaks, with maxima at 570 nm and 620 nm. Moreover, there are temporal changes observed in the fluorescence behavior of C12TBA-AgNCs. After 48 h in TRIS, the intensity of the peak at 570 nm slightly increases, whereas the emission at 620 nm clearly decreases. These changes might be attributed to structural modifications or interactions occurring within the nanocluster–DNA complex over time.

Both Tel22C12 and C12Tel22 DNA strands yield silver nanoclusters with emission maxima at 620 nm and 630 nm, respectively. The similarity in emission maxima suggests commonalities in the electronic transitions or energy levels associated with the fluorescence of these nanoclusters. Despite sharing similar emission maxima, significant differences exist in the fluorescence behaviors of the two systems. The silver nanoclusters synthesized on C12Tel22 exhibit higher fluorescence intensity compared to those on Tel22C12. The latter was confirmed by the emission quantum yield value, which is twice greater for C12Tel22 (ϕ = 0.002) than for Tel22C12 (ϕ = 0.001) in TRIS buffer. Moreover, a notable distinction lies in the stability of their fluorescence over time, as the silver nanoclusters formed on C12Tel22 DNA strands demonstrate enhanced stability in their fluorescence. The enhanced stability and higher fluorescence intensity observed in the C12Tel22-synthesized nanoclusters may be attributed to the specific interactions between the silver clusters and the DNA template, emphasizing the influence of subtle changes in the DNA sequence on the optical properties of the resulting nanomaterials.

**Figure 2:**
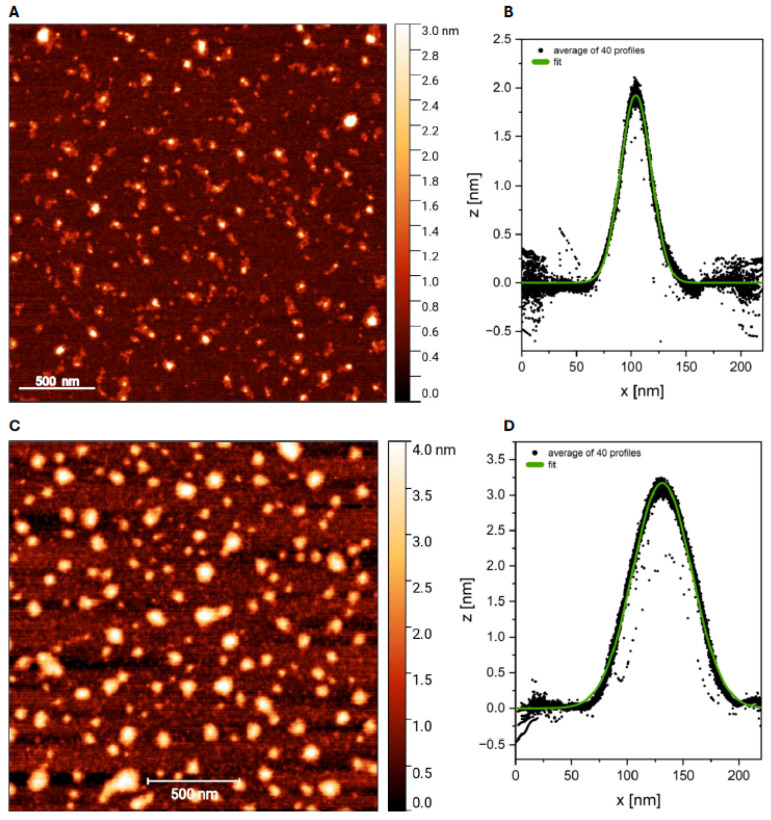
AFM image of C12TBA-AgNCs (**A**) in PBS buffer and corresponding height profile analysis (**B**). AFM image of C12Tel22-AgNCs (**C**) in PBS buffer and corresponding height profile analysis (**D**). The black dots represent the average of 40 profiles, and the green line indicates the fitted model.

Moreover, to confirm the relationship between nanocluster morphology and fluorescence, we imaged C12TBA-AgNCs in PBS and C12Tel22-AgNCs in PBS buffer using atomic force microscopy (AFM) (Figure 2 and Figure S4). The AFM images showed that the obtained silver nanoclusters are spherical in shape. Based on the analysis of the obtained profiles (*n* = 40), the dimensions of both investigated systems were determined, and the following values were obtained: C12TBA-AgNCs: 1.92 ± 0.15 nm and C12Tel22-AgNCs: 3.18 ± 0.17 nm. The second sample not only exhibits significantly larger dimensions but also appears to be more diverse in terms of its size and shape.

Based on the AFM images, both the Tel22C12-AgNCs and TBAC12-AgNCs samples demonstrated stronger tendencies toward aggregation than observed for the samples C12Tel22-AgNCs and C12TBA-AgNCs, which is visible in the images in Figures S5 and S6. However, examining the height of the individual objects was possible based on the images presented in Figures S5B and S6B. Measurements conducted on approximately 10 height profiles of Tel22C12-AgNCs showed an average height of 1.9 ± 0.42 nm. The TBAC12-AgNCs sample, analyzed using about 20 height profiles, exhibited a larger average height of 2.3 ± 0.35 nm. The TEM image confirmed that the sizes of the obtained silver nanoclusters are below 10 nm (Figure S7).

The distribution of hydrodynamic dimensions determined using the DLS method did not confirm any significant difference between C12TBA and C12Tel22 nanoclusters (Figure S8). The comparison of the size distributions for TBAC12 and Tel22C12 showed that TBAC12 nanoclusters are smaller than those of Tel22C12. In all solutions, a component originating from large aggregates was present, which could not be removed via a standard filtration procedure. However, its influence on the final result can be considered negligible. The dimensions of nanoclusters determined using this method are larger than those determined via AFM. However, such an effect was expected after taking into account hydration and mutual interactions.

### 2.2. Effect of Temperature on Stability of AgNCs Templated on C12 Integrated with G-Quadruplex-Forming Sequence

**Figure 3:**
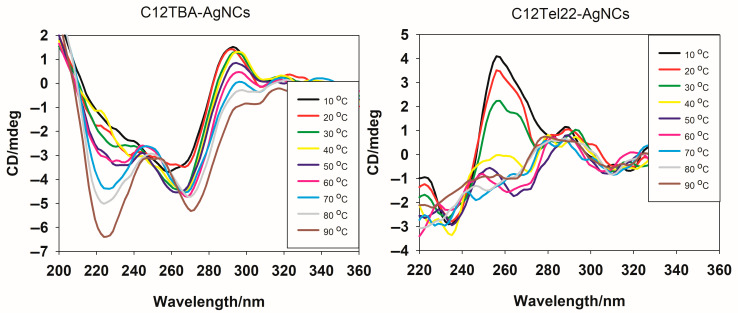
Circular dichroism spectra of C12TBA-AgNCs (**left**) and C12Tel22-AgNCs (**right**) in PBS. Conditions: DNA (2 mM), PBS buffer, pH = 7.4 (0.01 M), DNA:Ag^+^:BH_4_^−^ = 2:1:1, and temperature range from 10 °C to 90 °C, with 5 °C step.

The CD spectra of C12TBA-AgNCs and C12Tel22-AgNCs were taken from 10 °C onwards for the investigation of the temperature-dependent conformational behavior of the DNA-templated silver nanoclusters (Figure 3).

The CD spectra basically show a well-defined structure transition versus temperature for C12TBA-AgNCs. The spectra of C12TBA-AgNCs obtained at lower temperatures (10–30 °C) exhibit a negative band around 260 nm and a positive band in the vicinity of 295 nm, which are characteristic of G-quadruplex-like structures with anti-parallel topology. The increasing temperature leads to a gradual decrease in the intensity of the positive band, indicating a progressive disruption of the secondary structure. When reaching 90 °C, the spectrum shows a significant flat shape above 295 nm, indicating a loss of the ordered DNA conformation, most likely due to thermal denaturation. New negative peaks emerge at 225 nm and 275 nm, suggesting additional structural transitions or modifications within C12TBA-AgNCs as the temperature increases. The latter spectra remain the pattern observed previously after binding Ag^+^ preferentially with the DNA bases [34]. These observations highlight the thermal sensitivity of the C12TBA-templated nanocluster system and its dependence of structural integrity on optical activity. On the contrary, CD spectra of C12Tel22-AgNCs show a different thermal behavior. The spectra exhibit a marked positive band around 265–270 nm and a negative band in the vicinity of 245 nm at lower temperatures (10–30 °C), which indicates the presence of C12Tel22-AgNCs in parallel with G-quadruplex structures. The band intensity weakens with an increasing temperature, and at 50 °C, there is a completely different pattern of CD spectra observed, with a positive band at 295 nm, which is similar to that exhibited by the C12TBA-templated nanocluster system at this temperature. However, contrary to the C12TBA-AgNCs, we did not observe the strong negative peaks at 225 nm and 275 nm. What is most important, in both cases, is that the nanoclusters remain present in the solution below 40 °C, which means they can be tested *in cellulo*.

### 2.3. Influence of tC on Photophysical Properties of C12Tel22-AgNCs

To improve the fluorescence properties of DNA-templated nanoclusters, we decided to incorporate the fluorescent cytosine analog 1,3-diaza-2-oxophenoxazine (tC) into the cytosine-rich part of the DNA template.

1,3-Diaza-2-oxophenoxazine (tC) is a fluorescent nucleobase analogue that has gained attention in various fields of research, particularly in the study of nucleic acids [38]. Its unique structure allows it to form stable interactions with complementary bases, making it a valuable tool in oligonucleotide design [39,40].

In our research, we focused on synthesizing oligonucleotides that incorporate tC, aiming to explore how it influences the formation of nanoclusters. By examining these nanostructures, we sought to determine if tC could improve the fluorescence intensity, thermal stability, and resistance to enzymatic degradation. The choice of template was based on two parameters: fluorescence emission and their stability over time. First, we noticed that silver nanoclusters based on C12Tel22 DNA or Tel22C12 exhibit stronger and longer wavelength fluorescence emission than AgNCs on C12TBA or TBAC12 templates. Moreover, C12Tel22-AgNCs are more stable than Tel22C12-AgNCs.

**Figure 4:**
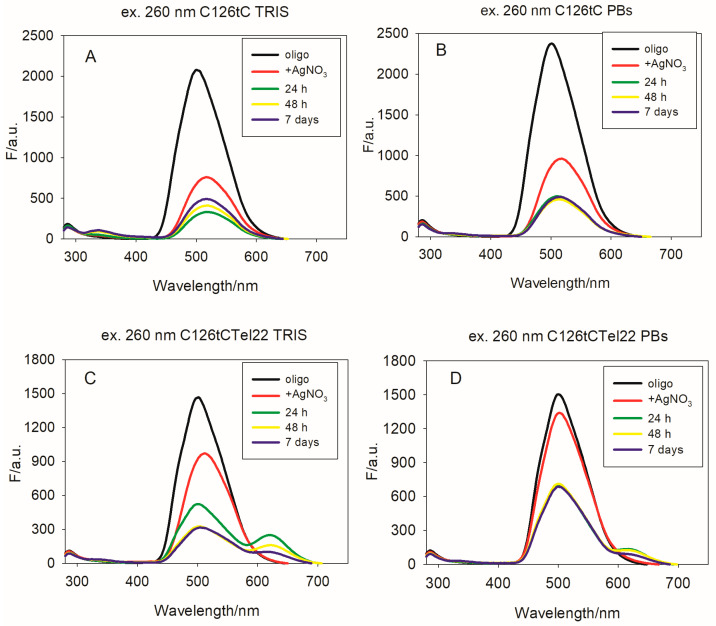
Fluorescence spectra of C126tC and C126tCTel22 at excitation wavelength of 260 nm in TRIS (**A**,**C**) and PBS (**B**,**D**) buffers. Conditions: DNA (2 µM), Tris-acetate (TRIS) buffer, pH = 7.4 (0.01 M), PBSbuffer, pH = 7.4 (0.01 M), and DNA:Ag^+^:BH_4_^−^ = 2:1:1. Ex bandwidth: 10 nm; Em bandwidth: 10 nm; response, 0.2 s; sensitivity: low; measurement range, 280–750 nm; scan speed, 1000 nm/min.

Therefore, for our purpose, we used the C12 oligonucleotide with tC at the sixth position from the 5′ end (called C126tC), as well as the C126tCTel22 template. In both cases, the excitation of tC-modified oligos by using 260 nm results in fluorescence with emission bands characteristic for tC, with λ_max_ = 500 nm (Figure 4). The emission spectra obtained during indirect excitation of the tC fluorophore (at 390 nm) are shown in Figure S9, and the corresponding excitation spectra, collected at λ emission = 505 nm, are shown in Figure S10. In the case of C126tC, the addition of silver ions before its reduction led to a slightly bathochromic shift of the emission band (λ_max_ moved to 509 nm) and presence of a weak intensity band, with λ_max_ = 348 nm (Figure 4A). These changes in the fluorescence spectra are caused by the formation of C126tC-AgNCs, which was confirmed by the CD and UV spectra (Figure S11).

In case of nanoclusters formed on the C126tCTel22 template in TRIS buffer (Figure S12), emission spectra exhibit a major peak situated at λ_max_ = 509 nm, as well as a clearly visible emission peak, with the λ_max_ around 620 nm (Figure 4). This emission can be directly excited at 575 nm, and in that case, it comes exclusively from nanoclusters. Moreover, the obtained emission spectra indicated that the strong fluorescence of C126tC as well as of C126tCTel22 coming from excited tC is red-shifted and quenched by the addition of Ag^+^. These results are in good agreement with previous results indicating that protonation of the tC fluorophore (tC-H^+^) is accompanied by a red-shift as well as a decrease in the fluorescence intensity [41]. The stability of C126tCTel22-AgNCs over time is better compared to identical nanoclusters without the incorporated tC moiety.

### 2.4. Detection of Potassium Ions Using Nanoclusters Based on C12Tel22 Template

**Figure 5:**
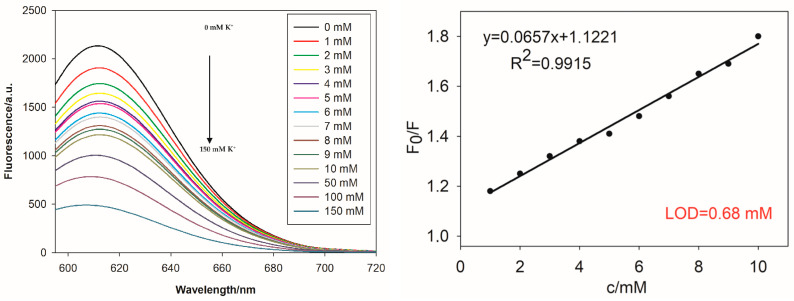
Fluorescence response of the C12Tel22-AgNCs in PBSbuffer at different K^+^ concentrations: the emission spectra of C12Tel22-AgNCs upon adding increasing concentrations of K^+^ ions (0–10 mM) (**left**) and the corresponding Stern–Volmer plot showing the K^+^ quenching effect on C12Tel22-AgNC emission spectra, with λ_max_ = 612 nm (**right**). Conditions: DNA (2 µM), PBS buffer, pH = 7.4 (0.01 M), DNA:Ag^+^:BH_4_^−^ = 2:1:1, and K^+^ 0–150 mM. Ex wavelength: 575 nm, Ex bandwidth: 10 nm; Em bandwidth: 10 nm; response, 0.2 s; sensitivity: medium; measurement range, 595–720 nm; scan speed, 1000 nm/min.

The studies on the effect of potassium cations on the spectral properties of C12Tel22-AgNCs and C126tCTel22-AgNCs were performed to select the potentially better sensors for potassium ions in extracellular conditions. First, to assess the analytical parameters of the proposed K^+^ ion detection system, the C12Tel22-AgNCs’ probe response (fluorescence intensity) was tested at different concentrations of potassium chloride in the concentration range of 0–150 mM in PBS buffer (Figure 5) as well as TRIS buffer (Figure S13). C12Tel22-AgNCs exhibit better stability in PBSbuffer than in TRIS (see Figure 1 and Figure S1). PBS buffer contains 137 mM NaCl, 2.7 mM KCl, 10 mM Na_2_HPO_4_, and 1.8 mM KH_2_PO_4_; therefore, it reflects the extracellular conditions in terms of sodium ion concentrations. As shown in Figure 5, the fluorescence intensity gradually decreases with an increasing K^+^ ion concentration. Based on the emission spectra, we constructed a Stern–Volmer plot, which illustrates the quenching effect of K^+^ ions on the emission of C12Tel22-AgNCs (Figure 5), where F_0_ means the maximum fluorescence intensity of C12Tel22-AgNCs without K^+^ ions, and F is the maximum fluorescence intensity after the addition of potassium ions. A good linear relationship of C12CTel22-AgNC fluorescence with the K^+^ concentration was observed in a range from 1 to 10 mM K^+^ in PBS buffer (R^2^ = 0.9915), and the limit of detection (LOD) was calculated to be 0.68 mM. The experiment was repeated in TRIS buffer containing 100 mM NaCl and led to the same LOD value of 0.68 mM (Figures S14 and S15). However, the LOD value in Tris-acetate buffer (TRIS) was estimated to be 1.5 mM (Figure S13).

**Figure 6:**
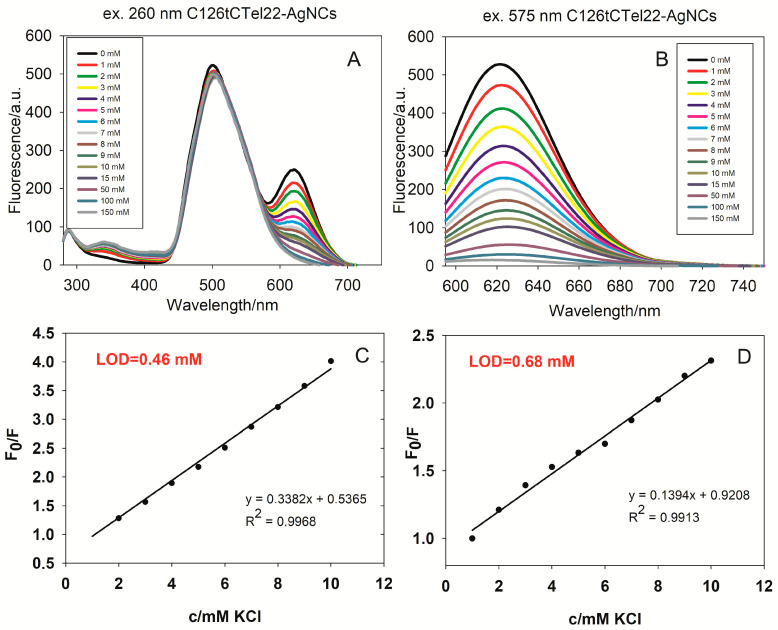
Fluorescence emission spectra of C126tCTel22-AgNCs during KCl titration, at excitation wavelengths of 260 nm (**A**) and 575 nm (**B**) in TRIS buffer. Corresponding Stern–Volmer plots, showing K^+^ quenching effect on emission spectra, with λ_max_ = 623 nm, in TRIS (**C**) and PBS (**D**) buffers, showing linear relationship in concentration ranges from 1 to 10 mM K^+^; conditions: DNA (2 µM), Tris-acetate (TRIS) buffer, pH = 7.4 (0.01 M), PBS buffer, pH = 7.4 (0.01 M), and DNA:Ag^+^:BH_4_^−^ = 2:1:1. Conditions: Ex bandwidth: 10 nm; Em bandwidth: 10 nm; response, 0.2 s; sensitivity: low; measurement range, 280–750 nm and 595–750 nm; scan speed, 1000 nm/min.

The two fluorescence spectra provided in Figure 6 show the behavior of C126tCTel22 silver nanoclusters (AgNCs) during titration with increasing concentrations of potassium chloride (KCl) in TRIS buffer. The first spectrum (A) shows the fluorescence intensity over a wavelength range from 300 nm to 750 nm, with excitation at 260 nm. There are two main fluorescence peaks observed: a prominent one around 500 nm and a secondary one around 630 nm. As the concentration of KCl increases (from 0 mM to 150 mM), only the intensity of the fluorescence peak at 630 nm changes notably. The second panel (B) displays the fluorescence intensity, focused on the 600–740 nm region, with excitation at 575 nm. As expected, a single fluorescence peak is observed around 630 nm, at which the fluorescence intensity decreases consistently with increasing KCl concentrations. The corresponding excitation spectra are shown in Figure S16. As previously, we constructed a Stern–Volmer plot, and we observed a good linear relationship of C126tCTel22-AgNC fluorescence with the K^+^ concentration in TRIS buffer over the range from 1 to 10 mM K^+^ (R^2^ = 0.9968), and the limit of detection (LOD) was calculated: 0.46 mM (Figure 6C). For C126tCTel22-AgNCs in PBS buffer (Figure S17), we also observed a good linear relationship in the range 0–10 mM, with R^2^ = 0.9913 and LOD = 0.68 mM (Figure 6D).The latter result is an identical to that obtained for C12CTel22-AgNCs in PBS.

### 2.5. Experiments in HeLa Cells

Although there have been promising developments in the use of silver nanoclusters for cell imaging, it is important to acknowledge that experimental outcomes can vary, and not all attempts at synthesis are successful. First, we wanted to gain experience in cell imaging experiments with DNA nanoclusters, and we made an effort to replicate an experiment outlined in the publication of Li et al. [42]. Therefore, we obtained nanoclusters based on the AS1411-T5 template (see Section 4.1 in Materials and Methods). Unfortunately, despite following the prescribed procedures [42], the silver nanoclusters synthesized on AS1411-T5 or on C12Tel22 templates did not exhibit the desired properties for effective cell imaging.

Next, in our exploration of silver nanoclusters for cell imaging, we incorporated a cholesterol anchor into the C12Tel22 template with the expectation that it would facilitate the binding of the obtained nanoclusters (ch-C12Tel22-AgNCs) to the cell membrane. The rationale behind this modification was to enhance the specificity of the silver nanoclusters for cellular imaging by targeting the lipid-rich cell membrane. The ability of cholesterol-modified ssDNA for spontaneous anchoring into the hydrophobic interior of lipid membranes was previously proven by Patolsky et al. [43].

**Figure 7:**
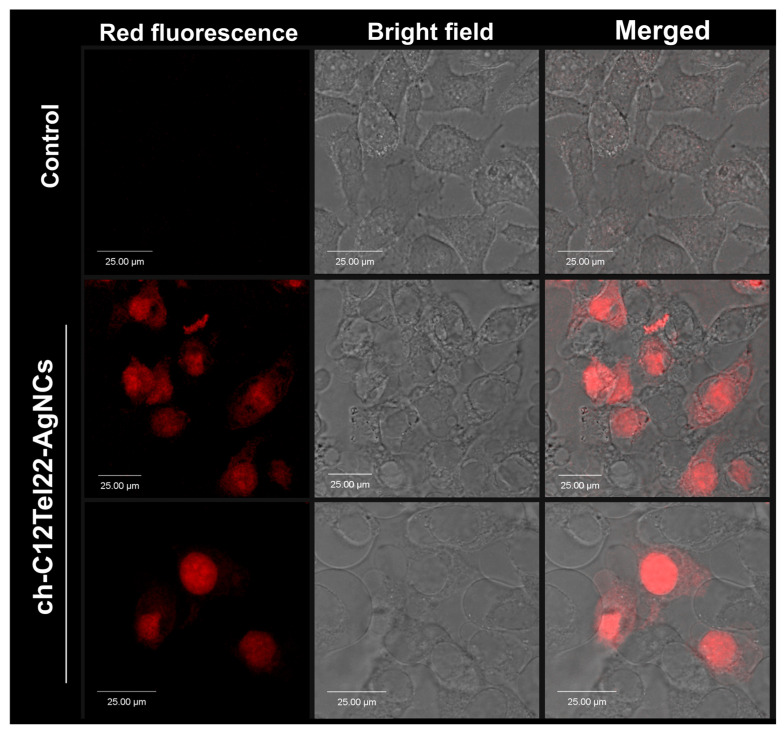
Confocal fluorescence imaging of HeLa cells loaded with 2 μM ch-C12Tel22-AgNCs for 10 min: fluorescence image (**left panel**) and white image (**middle panel**) and overlay of both images (**right panel**). Fluorescence emission filter: 600–700 nm; excitation wavelength: 595 nm.

However, contrary to our initial hypothesis, the silver nanoclusters, even with the cholesterol anchor, exhibited a propensity to penetrate into the cell rather than adhering primarily to the cell membrane (Figure 7). We supposed that a longer oligonucleotide probe than in case of our previous work [34] and its ability to form a G-quadruplex was the reason for this unanticipated outcome, which also suggests a unique interaction between the ch-C12Tel22-AgNCs and intracellular components.

Moreover, we tested the DNA-templated nanoclusters’ cytotoxicity in the Hela cell line according the MTT assay protocol. The cells were treated with varying concentrations of DNA and the corresponding DNA nanoclusters as follows: 250 nM, 1.0 µM, and 2.5 µM. The graphs of the percentage on viability of Hela cells against the concentrations of the tested nanoclusters (after 24 h of incubation) are presented in Figure S18. It is clearly shown that the formation of nanoclusters increases the cytotoxic activity against Hela cells compared to unmodified DNA templates. Interestingly, the incorporation of a cholesterol anchor causes slightly higher viability, which also decreases after nanocluster formation to a viability around 95% (for ch-C12Tel22-AgNCs).

**Figure 8:**
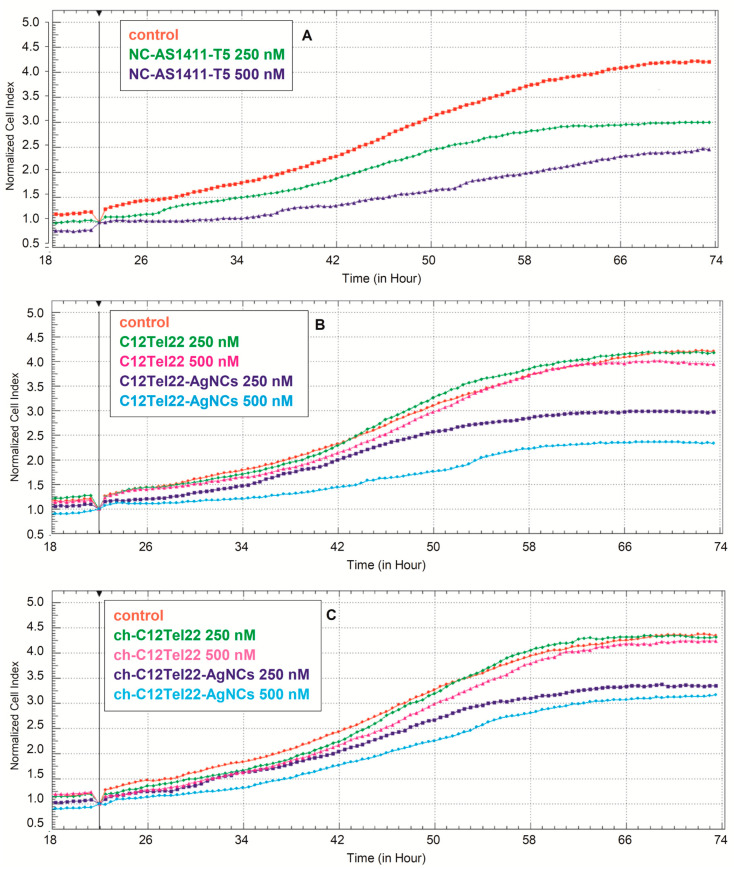
Real-time monitoring of the impact of oligonucleotides and silver nanoclusters on HeLa cell proliferation using the xCELLigence system. (**A**) NC-AS1411-T5; (**B**) C12Tel22 and C12Tel22-AgNCs; (**C**) ch-C12Tel 22 and ch-C12Tel22-AgNCs.

In addition, HeLa cell proliferation was monitored over a 96 h period using the xCELLigence real-time cell analysis system (Figure 8 and Table S1 in SI).

Oligonucleotide probes were added at 22 h at two concentrations as follows: 250 nM and 500 nM, as indicated by the transient drop in the Cell Index (CI) across all conditions (Figure 8). The untreated control group (red) exhibited continuous proliferation, reaching a CI of approximately 3.0 at 48 h (Table S1). Treatment with nanoclusters: 250 nM NC-AS1411-T5 (Figure 8A, green) and 500 nM (blue) significantly reduced the Cell Index values, demonstrating a dose-dependent antiproliferative effect. The free C12Tel22, especially at lower doses, did not affect the change in proliferation (Figure 8B, green and magenta). On the other hand, the nanocluster-conjugated form (C12Tel22-AgNCs) caused a reduction in proliferation (Figure 8B, blue), and the higher concentration had the strongest suppressive effect (Figure 8B, cyan). The same trend was noticed for ch-C12Tel22 (Figure 8C); the free form did not cause reduction in cell growth, particularly at lower doses (green and magenta), while its nanocluster conjugation exerted opposite effect in a concentration-dependent manner. Higher concentrations of the conjugated probes resulted in distinct inhibition of cell proliferation (Figure 8C, blue and cyan).

In summary, the proliferative response was negligible for oligonucleotides C12Tel22 and ch-C12Tel22 with a concentration of 250 nM and 500 nM, while NC-AS1411-T5 and C12Tel22-AgNCs, especially at higher concentrations, inhibited cell growth clearly. It is worth emphasizing that incorporation the cholesterol moiety into C12Tel22-AgNCs decreased their antiproliferative effect, significantly at higher concentration.

## 3. Discussion

The intriguing aspect of the fluorescence behavior observed in the emission spectra lies in the sensitivity of nanocluster formation to the specific placement of the C12 moiety within the DNA sequence, as well as the buffer used during synthesis. Notably, the nearly identical DNA structures of TBAC12 and C12TBA or Tel22C12 and C12Tel22, with the sole distinction being the position of C12, located at the 3′ end or at the 5′ end of oligo template, yield different silver nanoclusters. This subtle alteration in the placement of the C12 moiety leads to distinctive silver nanocluster formations and subsequent variations in fluorescence characteristics. Despite the structural similarity in the overall DNA composition, the specific positioning of C12 has a discernible impact on the resulting nanocluster properties. Such sensitivity underscores the intricate relationship between the DNA template and the silver nanocluster assembly, where subtle changes in the DNA sequence can yield marked differences in the optical properties of the formed nanoclusters.

The fact that the location of C12, whether at the 3′ or 5′ end, results in different silver nanoclusters with varying fluorescence profiles adds a layer of complexity to the under-standing of these nanostructures. This observation may be attributed to the influence of the local DNA environment on the nucleation and growth of the silver nanoclusters, emphasizing the importance of precise control over the DNA template for tailoring the properties of the resulting nanomaterial.

The divulged results, thus, highlights the important role of the C12 modification site on the structural dynamics and thermal stability of the DNA–silver nanocluster system. The variation in CD spectra and temperature-dependent profiles distinctly suggest the influence of the sequence orientation on the folding and stabilization of the DNA templates, which, in turn, affects nanocluster construction and the optical properties.

The reduction in fluorescence intensity with a higher KCl concentration indicates a likely quenching effect, potentially due to changes in the nanocluster structure or environment in the presence of potassium ions. As each system contains a G-quadruplex-forming sequence, it seems probable that the formation of G-quadruplexes upon K^+^ addition causes structural changes, which impact the nanoclusters’ structure, which is demonstrated by the decrease in their emission.

The cholesterol-bearing silver nanoclusters happened to exhibit negligible cytotoxicity; unfortunately, they easily get internalized instead of anchoring into the cell membrane. As we delve deeper into the intricacies of this unexpected result, we remain committed to refining our understanding of the interaction dynamics between the modified silver nanoclusters and cellular components. This knowledge should not only contribute to the development of effective imaging agents but also open new avenues for the design and optimization of nanomaterials tailored for specific cellular targets.

In summary, the unique optical properties, biocompatibility, and tunable fluorescence of silver nanoclusters make them promising candidates for cell imaging applications.

## 4. Materials and Methods

### 4.1. Materials

Most oligonucleotides were purchased as indicated in Table 1. The concentrations of the nucleotides were calculated based on the absorbance at 260 nm, measured at a neutral pH and at 85 °C, using extinction coefficients calculated from the published values of molar absorptivity of nucleotides [44]. Dimethylarsinicacid sodium salt trihydrate (≥98%), silver (I) nitrate (AgNO_3_, ≥99.0%), sodium borohydride (NaBH_4_, ≥98%), sodium chloride (NaCl, ≥99.0%), and potassium chloride (KCl, ≥99.0%) were purchased from Sigma-Aldrich. Tris (Hydroxymethyl)Aminomethane (Tris) (BioUltraPure) and Phosphate-Buffered Saline (PBS tablets) were purchased from BioShop. All stock solutions were prepared using MilliQ-type purified water with the Ultrapure Water Simplicity UV system and autoclaved using the Classic Prestige Medical Autoclave.

### 4.2. Methods

#### 4.2.1. DNA-Ag Nanocluster Synthesis

Silver nanoclusters were synthesized following the procedure described in our previous work [22]. The synthesis was performed using 2 µM solution of DNA oligonucleotides dissolved in PBS (10 mM, pH = 7.4) or TRIS (10 mM, pH = 7.4). The obtained DNA-AgNCs were further stored in the dark at 4 °C.

#### 4.2.2. Fluorescence Spectroscopy

Fluorescence spectra (emission or excitation) were recorded on the Jasco FP-8200 spectrofluorometer (Tokyo, Japan) equipped with a Peltier accessory set at 25 °C and were carried out using a 0.4 × 1 cm quartz cuvette containing 1 mL of sample. Spectra were recorded with a recording speed of 1000 nm/min, with excitation and emission slits equal to 10 nm. The recording range of the fluorescence spectra and their excitation and emission wavelengths are provided in the captions of figures.

#### 4.2.3. Circular Dichroism (CD) Spectroscopy

CD spectra were measured on a Jasco J-820 Spectropolarimeter (Tokyo, Japan) equipped with a PTC-423L temperature controller set at 25 °C. Each spectrum was the average of three repeated scans recorded from 200 to 400 nm with a 10 mm quartz cell at a scan speed of 500 nm/min. The corresponding buffer (10 mM Tris–acetate or 10 mM PBS) was used as a blank solution and was subtracted from the average scan for each sample.

#### 4.2.4. UV-Vis Absorption Spectroscopy

UV-Vis absorption spectra were measured with a Jasco V-750 spectrophotometer (Tokyo, Japan) equipped with a Peltier-type temperature controller set at 25 °C. Absorption spectra were recorded in the spectrum range of 220–800 nm with a recording speed of 500 nm/min.

#### 4.2.5. Studies upon Metal Cation Effect

The metal cation effect was studied in the concentration range of 0–0.15 M. The typical experiment consisted of successive additions of small portions of a concentrated solution of 3 M KCl salt to 1 mL of DNA-AgNCs prepared 24 h earlier. After each addition, the solution was stirred, followed by thermal equilibration and after 3 min, fluorescence spectra were measured.

#### 4.2.6. Oligonucleotide Synthesis

Syntheses were performed on a DNA/RNA Synthesizer H-6 (K&A Laborgeraete, Schaafheim, Germany) applying a 0.2 μmol protocol, commercial ultramild phosphoramidites, tC-CE phosphoramidite, and CPG supports. In case of oligonucleotides with tC, for tC phosphoramidite, the coupling time was extended to 3 min.

#### 4.2.7. Deprotection and Purification of Oligonucleotides

The support with the oligonucleotide was placed into a vial, 1 mL of ammonium hydroxide was added, and the vial was closed tightly. After 24 h at 25 °C, the solid was filtered off and washed with methanol, and the filtrates were evaporated to dryness. The residue was dissolved in 0.1 M ammonium acetate (1 mL) and passed through a NAP 25 column. The column was washed with the same solution. Fractions that contained oligonucleotides were combined and evaporated, dissolved in H_2_O/Acetonitrile, 95/5 *v*/*v* (1 mL), and purified via HPLC (XBridge Oligonucleotide BEH C18 OBD Prep Column; 2.5 μm, 10 × 50 mm; phase A = 0.1 M TEAA; B = 0.1 M TEAA/Acetonitrile; 80/20 *v*/*v*; flow rate 1.5 mL/min; gradient 0–15% B in 30 min). Combined fractions were concentrated to approx. 0.5 mL and desalted via passaging through an Amicon Ultra Centrifugal Filter, 3 kDa MWCO. HPLC analysis of the obtained oligonucleotides was performed (XBridge OST C18 Column; 2.5 μm, 4.6 × 50 mm; phase A = 0.1 M TEAA; B = 0.1 M TEAA/Acetonitrile; 80/20 *v*/*v*; flow rate 0.5 mL/min; gradient 0–30% B in 30 min).

#### 4.2.8. Atomic Force Microscope (AFM) Measurements

The surface topography of the samples was investigated using an atomic force microscope (Nanowizard IV, Bruker, formerly JPK, Berlin, Germany) operating in tapping mode. Sample preparation involved deposition on thoroughly cleaned mica surfaces. Before the sample application, the mica surface was functionalized with 10 mM CaCl_2_ solution for 5 min and then rinsed with Millipore-grade water. The samples were then loaded onto the functionalized mica surface and incubated for 2 min, followed by another rinsing step with Millipore water. The prepared samples were left to air-dry at room temperature before AFM imaging. All measurements were performed using HQ:NSC35/No Al probes (Innovative Solutions Bulgaria Ltd., Sofia, Bulgaria) with 3 different soft-tapping mode AFM Cantilevers (radius < 8 nm), with spring constants of 0.5, 1, and 2 N/m and resonant frequencies of 65, 90, and 130 kHz, respectively. AFM images were recorded with a resolution of 512 × 512. Images were processed and analyzed using JPK Data Processing and Gwyddion v2.67 software (http://gwyddion.net/, accessed on 15 May 2025). Samples were prepared 24 h before measurement, stored in a refrigerator, and measured immediately after temperature stabilization without further purification.

#### 4.2.9. Dynamic Light Scattering (DLS) Measurements

DLS measurements were performed using the particle analyzer Litesizer™ 500 from Anton Paar (Anton Paar, Graz, Austria) equipped with a 658 nm diode laser with a power of 40 mW. The temperature of the samples during the DLS measurement was set at 20 °C. Detection of scattered light at an angle of 90° was automatically adjusted by the Litezer optical system. The particle size distribution was measured in a low-volume quartz cuvette. Kalliope Professional (Anton Paar, version 3.2.5) software was used to collect and analyze the data. All measurements were performed in PBS buffer (pH 7.4). Samples were prepared 24 h before measurement, stored in a refrigerator, and measured immediately after temperature stabilization without further purification.

#### 4.2.10. Transmission Electron Microscopy (TEM) Microscope Measurements

TEM measurements were collected and processed at the SOLARIS National Synchrotron Radiation Centre (Kraków, Poland). The cryoEM images were collected using the Glacios Cryo-TEM microscope (nominal magnification 150,000× *g*) operated with a 200 kV X-FEG source (Thermo Fisher Scientific, Waltham, MA, USA). The C12Tel22-AgNCs solution (volume 3 µL) was applied without dilution onto grids (carbon/Cu, mesh 200) and vitrified using Vitro-bot MkIV (Thermo Fisher Scientific, Waltham, MA, USA).

#### 4.2.11. Fluorescence Imaging Experiments Using Confocal Microscopy

For confocal microscopy analysis, the HeLa cells were seeded at a density of 1.2 × 10^5^ cells per well in 4-chamber glass-bottom cell culture dishes (Grenier Bio-One, Monroe, NC, USA) and cultured in RPMI 1640 medium (Sigma, St. Louis, MO, USA) supplemented with 10% (*v*/*v*) fetal bovine serum (FBS) (Gibco, Waltham, MA, USA), 1% RPMI 1640 vitamin solution (Sigma, St. Louis, MO, USA), and 1% antibiotic antimycotic solution (Sigma, St. Louis, MO, USA) at 37 °C under a 5% CO_2_ atmosphere and incubated until 80–90% confluency. Then, the cells were treated with ch-C12Tel22-AgNCs at a final concentration of 2 µM for 10 min; the untreated cells were used as a negative control. After the incubation period, the cells were washed twice with phosphate-buffered saline (PBS), placed in Fluoro Bright DMEM (Thermo Fisher Scientific, Waltham, MA, USA), and immediately examined under a Leica TCS SP5 II confocal microscope at 37 °C using an environmental cell culture chamber and an HC PL APO 63×/1.2 water-immersion objective. Sequentially scanned Z-stack images were collected at an excitation/emission wavelength of 595/600–700 nm for fluorescence detection. Image acquisition was controlled using Leica LAS AF 2.7.3 software, and image processing was performed using Leica LAS X 3.3.3 software with the 3D deconvolution module. Z-projections were generated from the Z-stacks using the max-intensity option in LAS X software.

#### 4.2.12. Real-Time Cell Proliferation Assay Using the xCELLigence System

Cell proliferation analysis was performed using the xCELLigence system (Roche, South San Francisco, CA, USA). Briefly, 100 µL of supplemented medium was added to each well of 16-well E-plates to 226 to measure the baseline values. Next, cells were seeded in an additional 100 µL of medium at a final density of 1 × 10^3^ cells per well and allowed to settle at the bottom of the wells at 37 °C and 5% CO_2_ for 30 min. After 22 h, the cells were treated with oligonucleotides or silver nanoclusters at final concentrations of 250 and 500 nM. Untreated control cells were cultured in supplemented medium only. Real-time cell proliferation was monitored using RTCA Software 1.2.1 for 96 h at 30 min intervals and expressed as a Normalized 232Cell Index (NCI). For each condition, the experiment was performed in triplicate biological replicates. Vertical lines in the graphs indicate the normalization time point (i.e., the time of treatment with oligonucleotides or silver nanoclusters). The NCI was calculated automatically by the software using the formula NCI = CI(t)/CI(t_0_), where NCI is the Normalized Cell Index, CI(t) is the Cell Index at time t, and CI(t_0_) is the Cell Index at the normalization time point (t_0_, the time of treatment).

## 5. Conclusions

In presented work, we continue our studies on fluorescent silver nanoclusters formed on bifunctional oligonucleotides consisting of a cytosine-rich DNA template (C12) attached to a G-quadruplex (GQ)-forming sequence, derived from TBA or human telomeric (Tel22) sequence, as a potential potassium ion probe. The GQ domain is responsible for potassium ion complexation, as G-quadruplexes exhibit a higher binding preference for potassium over sodium ions. Our studies indicate that the location of the C-rich domain (3′ or 5′ end of GQ), mainly responsible for silver complexation, has an undeniably important impact on the fluorescence properties and stability of the obtained nanoclusters. Therefore, dual-emitting clusters of Ag atoms can be successfully obtained on a bifunctional oligonucleotide consisting of a cytosine-rich DNA template (C12) attached to 3′ end of a GQ-forming sequence (TBA or Tel22). In contrary, the location of the C12 domain at the 5′ end of the GQ leads to dual-emitting clusters only in the case of C12TBA. For C12Tel22, we obtained formation only red-emitting nanoclusters (with a fluorescence maximum at 620 nm) and twice-stronger fluorescence in terms of quantum yield. The introduction of the fluorescent cytosine analog tC led to nanoclusters with slightly red-shifted emission and increased stability compared to the unmodified C12Tel22 template of the nanoclusters. The limit of detection for the C12Tel22-AgNC as well as C126tCTel22-AgNCs K^+^ probes in extracellular-mimicking conditions is at the value of 6.8 × 10^−4^ M. For us, it was important to develop systems exhibiting a linear working range for K^+^ detection from 1 to 10 mM in the presence of 150 mM Na^+^, which covers a clinically important concentration region of K^+^ (3.5 to 5.5 mM) under extracellular conditions. The sensitivity of the proposed systems could be improved by changing the G-quadruplex-forming sequence or finding another type of aptamer able to bind potassium ions. The integration of C12Tel22-AgNCs with a cholesterol moiety was performed in order to anchor it to the living cell membrane. However, the observed internalization of the cholesterol-modified C12Tel22-AgNCs probe rendered it impossible to investigate its ability to serve as bioimaging tool for visualizing the transmembrane transport of potassium cations. Further studies are needed to understand how and why the nanoclusters are internalized into the cells. As research in this field continues, silver nanoclusters may play a crucial role in advancing our understanding of cellular processes and contribute to the development of novel diagnostic and therapeutic strategies.

## Figures and Tables

**Table 1:** The DNA oligonucleotide sequences used in this research.

Name	Oligonucleotide Sequence	Company
Tel22C12	5′-AGG GTT AGG GTT AGG GTT AGG GCC CCC CCCCCC C-3′	Genomed (Warszawa, Poland)
TBAC12	5′-GGT TGG TGT GGT TGG CCC CCCCCC CCC-3′	Genomed (Warszawa, Poland)
C12Tel22	5′-CCC CCC CCCCCC AGG GTT AGG GTT AGG GTT AGG G-3′	Genomed (Warszawa, Poland)
C12TBA	5′-CCC CCC CCCCCC GGT TGG TGT GGT TGG-3′	Genomed (Warszawa, Poland)
C126tC	5′-CCC CCtC CCC CCC-3′	Synthesized by us
C126tCTel22	5′-CCC CCtC CCC CCC AGG GTT AGG GTT AGG GTT AGG G-3′	Eurogentec (Seraing Belgium)
ch-C12Tel22	5′-ch-CCC CCC CCCCCC AGG GTT AGG GTT AGG GTT AGG G-3′	Eurogentec (Seraing Belgium)
AS1411-T5	5′-CCC CCC CCCCCC TTTTTGGTGGTGGTGGTGTGGTGGTGGTGG-3′	Genomed (Warszawa, Poland)

## Data Availability

Data are provided in the manuscript.

## Supplementary Materials

The following supporting information can be downloaded at: https://www.mdpi.com/article/10.3390/ijms26104914/s1.
