# Supplementary material for: Studies upon Fluorescent Modulation of Silver Nanoclusters Formed on Bifunctional DNA Template
Source: Int J Mol Sci. 2025 May 20;26(10):4914. doi: 10.3390/ijms26104914 (PMC12112475; doi:10.3390/ijms26104914)
Supplement: Supplementary file 1 [file ijms-26-04914-s001.zip › ijms-3590226-supplementary-conversion.pdf]

Supplementary Materials

Studies Upon Fluorescent Modulation of Silver Nanoclusters Formed on Bifunctional DNA Template

Patrycja Filipczuk [1], Agnieszka Fedoruk-Wyszomirska [2], Joanna Nowak-Karnowska [1,*], ZuzannaPietralik-Molińska [3], Ewa Banachowicz [3], Maciej Kozak [3,4] and Anna Dembska [1,*]

[1] Faculty of Chemistry, Adam Mickiewicz University, Uniwersytetu Poznańskiego 8, 61-614 Poznań, Poland, patrycja.filipczuk@amu.edu.pl (P.F.), j.nowak@amu.edu.pl (J.N.-K.), aniojka@amu.edu.pl (A.D.)
[2] Institute of Human Genetics Polish Academy of Sciences, ul. Strzeszyńska 32, 60-479 Poznań, Poland, agnieszka.fedoruk-wyszomirska@igcz.poznan.pl (A.F.-W.)
[3] Faculty Department of Biomedical Physics, Faculty of Physics and Astronomy, Adam Mickiewicz University, Uniwersytetu Poznanskiego 2, 61-614 Poznan, Poland, zuzanna.pietralik@amu.edu.pl (Z.P.-M.), ewa.banachowicz@amu.edu.pl (E.B.), maciej.kozak@amu.edu.pl (M.K.)
[4] National Synchrotron Radiation Center SOLARIS, Jagiellonian University, Czerwone Maki 98, 30-392 Krakow, Poland
* Correspondence: j.nowak@amu.edu pl and aniojka@amu.edu.pl; Tel.: +48-61-829-1771

No.	Title
Figure S1	Absorption spectra of TBAC12-AgNCs, Tel22C12-AgNCs, C12TBA-AgNCs, and C12Tel22-AgNCs over time.
Figure S2	Emission spectra upon excitation at 570 nm of Tel22C12-AgNCs and C12Tel22-AgNCs in PBS buffer.
Figure S3	Emission spectra upon excitation at 260 nm of TBAC12-AgNCs, C12TBA-AgNCs, Tel22C12-AgNCs, and C12Tel22-AgNCs in TRIS buffer.
Figure S4	AFM topographic image of a large area of the C12TBA-AgNC sample.
Figure S5	AFM images of fluorescent Tel22C12-AgNCs in PBS buffer.
Figure S6	AFM images of fluorescent TBAC12-AgNCs in PBS buffer.
Figure S7	Exemplary transmission image of C12Tel22-AgNC sample recorded using Glacios Cryo-TEM Microscope.
Figure S8	Examples of the dynamic light scattering (DLS) data on the distribution of the relative intensity of scattered light by nanocluster size for C12TBA-AgNCs (blue) and C12Tel22-AgNCs (yellow).
Figure S9	Fluorescence emission spectra of C126tC-AgNCs and C126tCTel22-AgNCs at excitation wavelength of 390 nm in TRIS (a, c) and PBS (b,d) buffers.
Figure S10	Fluorescence excitation spectra of C126tC-AgNCs and C126tCTel22-AgNCs at emission wavelength of 505 nm in TRIS (a, c) and PBS (b, d) buffers.
Figure S11	Circular dichroism and absorbance spectra of C126tC over time in TRIS buffer.
Figure S12	Absorbance and circular dichroism spectra of C126tCTel22-AgNCs over time in TRIS buffer.
Figure S13	Changes over time in fluorescence spectra of C126tCTel22-AgNCs in TRIS excited at 260 nm (a) and 390 nm (b).
Figure S14	Fluorescence response of the C12Tel22-AgNCs in TRIS buffer at different K^+ concentrations: the emission spectra of C12Tel22-AgNCs upon adding increasing concentrations of K^+ ions (0–150 mM) (left) and corresponding Stern–Volmer plot.

Figure S15	Fluorescence response of the C12Tel22-AgNCs in TRIS buffer containing 100 mM NaCl at different K^+ concentrations: the emission spectra of C12Tel22-AgNCs upon adding increasing concentrations of K^+ ions (0–150 mM) (left) and corresponding Stern–Volmer plot.
Figure S16	Fluorescence excitation spectra of the C12Tel22-AgNCs at emission wavelength of 625 nm in TRIS buffer containing 100 mM NaCl upon adding increasing concentrations of K^+ ions (0–150 mM).
Figure S17	Fluorescence excitation spectra of the C12Tel22-AgNCs at emission wavelength of 500 nm (left) and 620 nm (right) in TRIS buffer upon adding increasing concentrations of K^+ ions (0–150 mM).
Figure S18	Fluorescence emission spectra of C126tCTel22-AgNCs during KCl titration, at excitation wavelengths of 260 nm (left) and 575 nm (right) in PBS buffer.
Figure S19	Effect of the tested oligonucleotides and silver nanoclusters (concentration range 0.25–2.5 μM) on the viability of Hela cells after 24 h incubations. Data presented as the mean ± SD.
Table S1	Changes in the Cell Index (CI) from 22.5 h to 96 h in HeLa cells treated with G-rich oligonucleotides and their silver nanocluster conjugates.

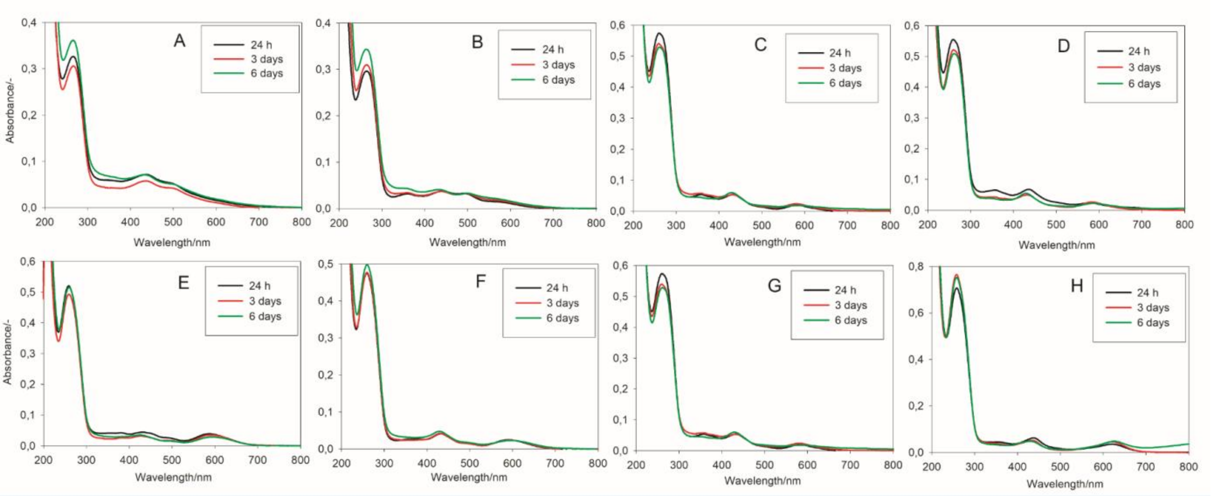

Figure S1. Absorption spectra of TBAC12-AgNCs (a,b), C12TBA-AgNCs (c,d), Tel22C12-AgNCs (e,f), and C12Tel22-AgNCs (g,h) over time. Conditions: DNA (2 μM), PBS buffer, pH = 7.4 (0,01 M), and DNA:Ag^{+}:BH_{4-} = 2:1:1.

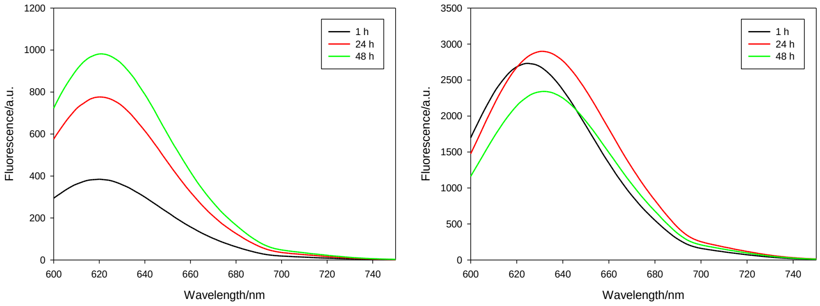

Figure S2. Emission spectra upon excitation at 570 nm of Tel22C12-AgNCs (left) and C12Tel22-AgNCs (right); conditions: DNA (2 μM), PBS buffer, pH = 7.4 (0,01 M), and DNA:Ag^{+}:BH_{4-} = 2:1:1. Ex bandwidth of 10 nm, em bandwidth of 10 nm, response of 0.2 sec, and sensitivity: medium.

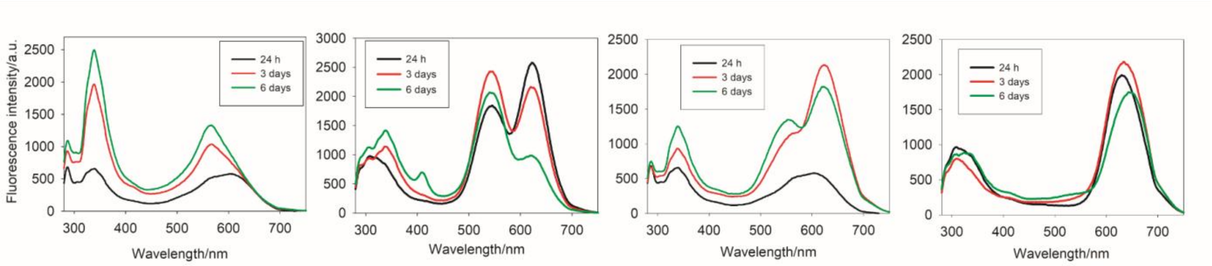

Figure S3. Emission spectra upon excitation at 260 nm of TBAC12-AgNCs (a), C12TBA-AgNCs (b), Tel22C12-AgNCs (c), and C12Tel22-AgNCs (d); conditions: DNA (2 μM), Tris-acetate, pH = 7.4 (0,01 M), and DNA:Ag^{+}:BH_{4-} = 2:1:1. Ex bandwidth of 10 nm, em bandwidth of 10 nm, response of 0.2 sec, and sensitivity: medium.

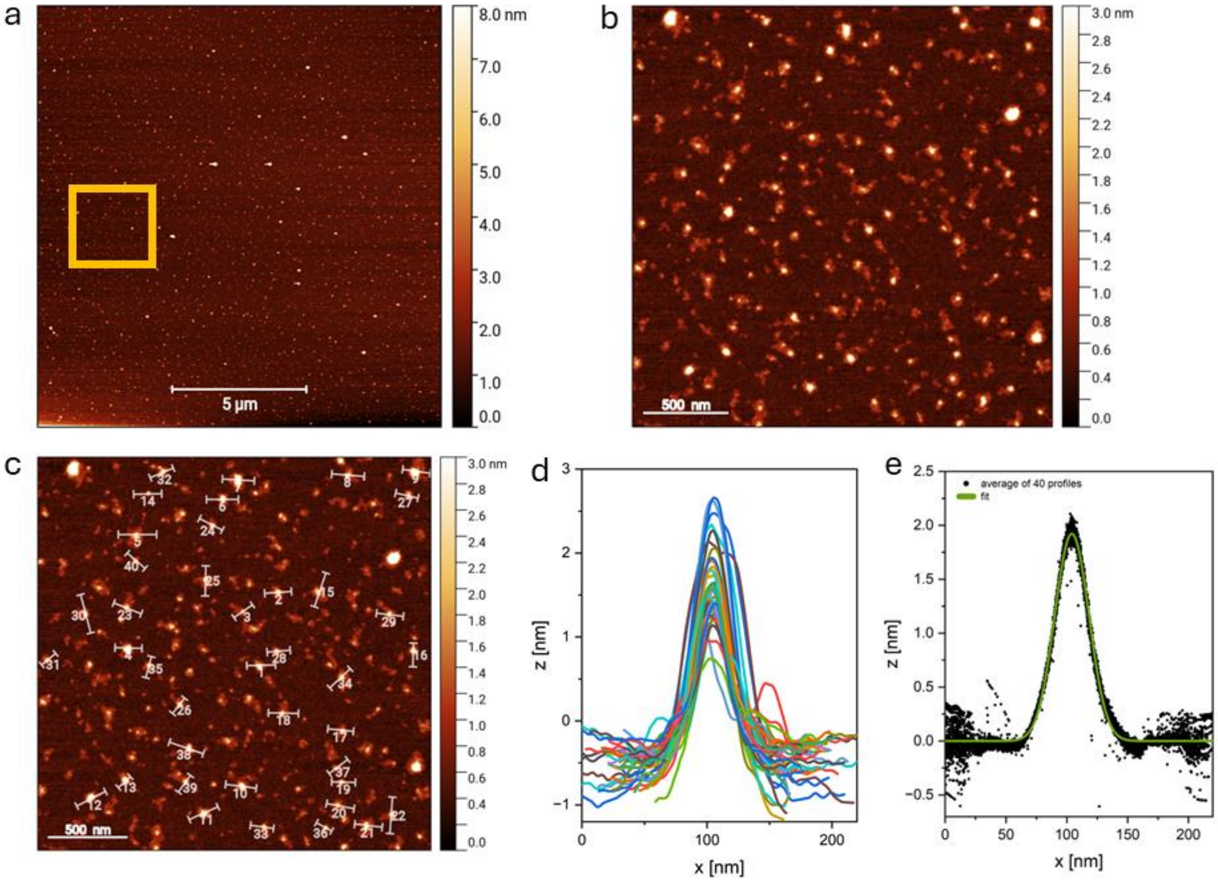

Figure S4. AFM topographic image of a large area of the C12TBA-AgNCs sample (a) with a marked region indicating the location of the higher-resolution scan presented in (b). The same area as shown in (b) but with marked positions of 40 profile lines used for height analysis (c). Height profile plots (d) corresponding to the 40 individual line scans marked in panel (c). Statistical analysis of the height profiles showing the average of all 40 profiles (black dots) with a fitted Gaussian model (green line) (e).

The image in Fig S4 (a) provides an overview of the nanoparticle distribution across the sample surface, whereas the higher-resolution AFM scan reveals detailed surface features and individual nanoparticle structures of the C12TBA-AgNC sample. Based on this image, profiles were selected (c) to characterize the representative nanoparticle features across the sample. This analysis provides quantitative information about the average nanoparticle dimensions.

a) b)

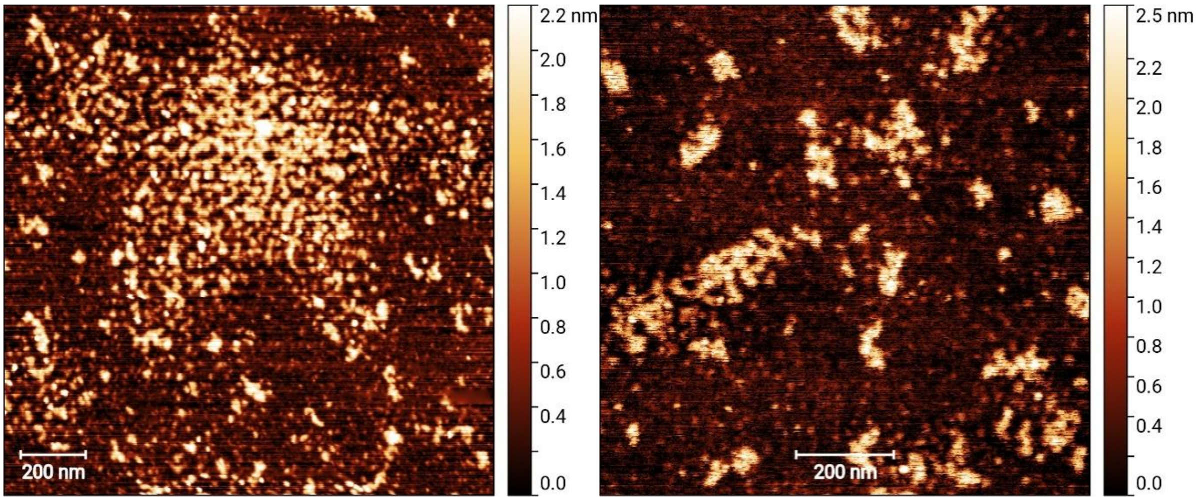

Figure S5. AFM images of fluorescent Tel22C12-AgNCs in PBS buffer.

a) b)

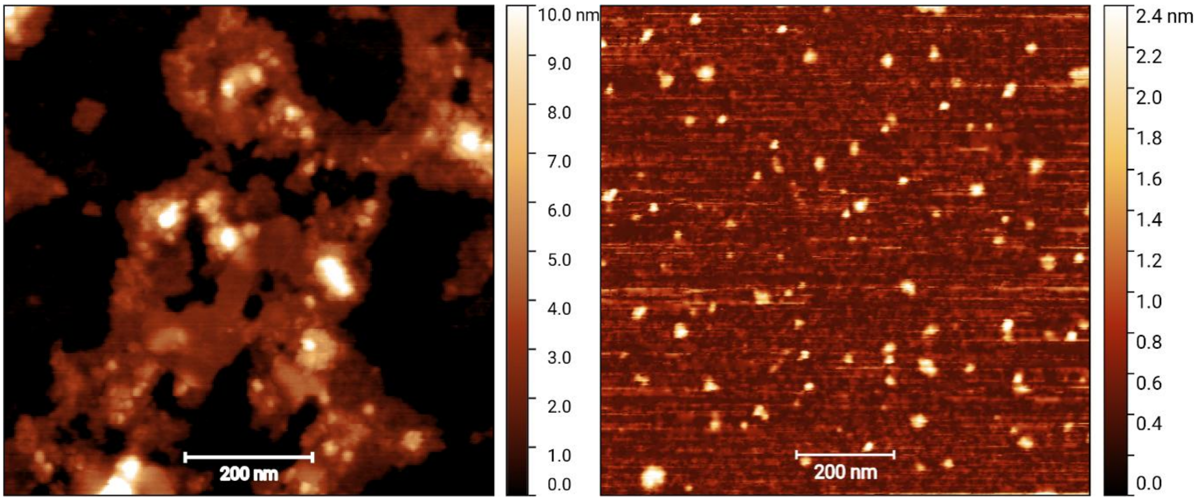

Figure S6. AFM images of fluorescent TBAC12-AgNCs in PBS buffer.

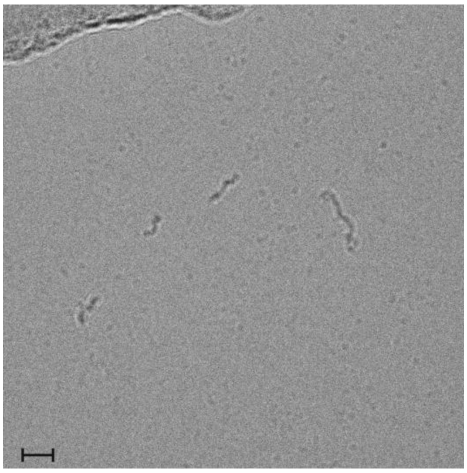

Figure S7 Exemplary transmission image of DNA-Q-C12-Tel22 sample recorded using Glacios Cryo-TEM Microscope (Thermo Fisher Scientific). Scale bar on the image represents 20 nm size.

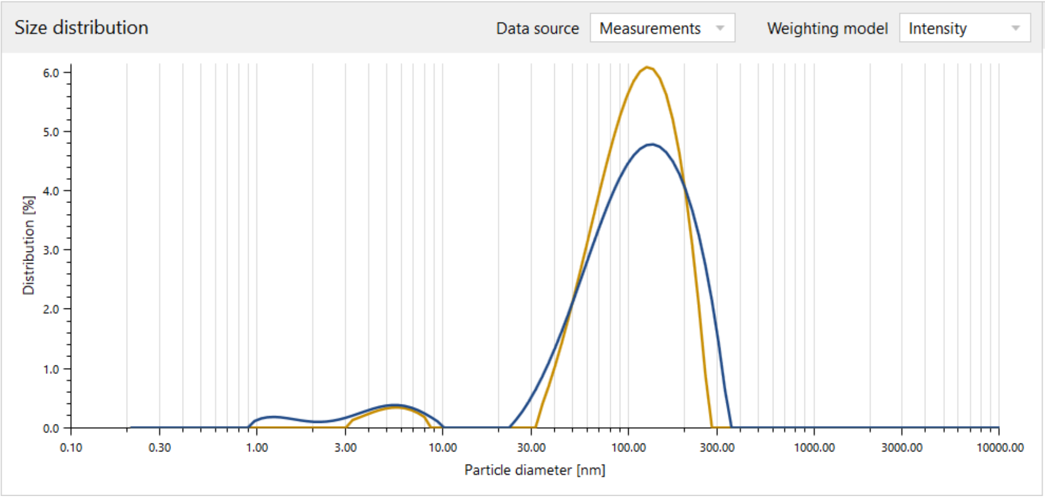

Figure S8. Examples of the dynamic light scattering (DLS) data on the distribution of the relative intensity of scattered light by nanocluster size for C12TBA-AgNCs (blue) and C12Tel22-AgNCs (yellow).

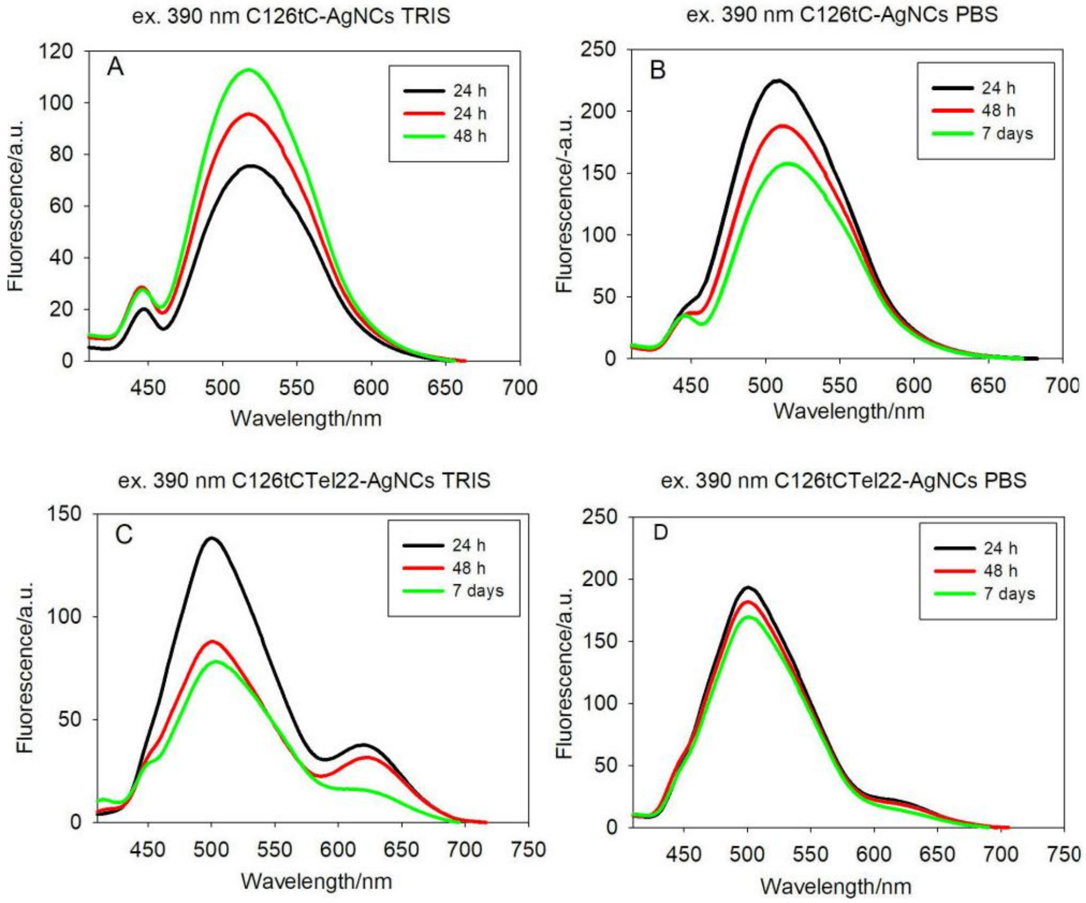

Figure S9. Fluorescence emission spectra of C126tC-AgNCs and C126tCTel22-AgNCs at excitation wavelength of 390 nm in TRIS (a, c) and PBS (b, d) buffers. Conditions: DNA (2 μM), Tris-acetate (TRIS) buffer, pH = 7.4 (0.01 M), PBS buffer, pH = 7.4 (0.01 M), and DNA:Ag^+:BH_4^- = 2:1:1. Ex bandwidth: 10 nm; Em bandwidth: 10 nm; response, 0.2 sec; sensitivity: low; measurement range, 280-750 nm; scan speed, 1000 nm/min.

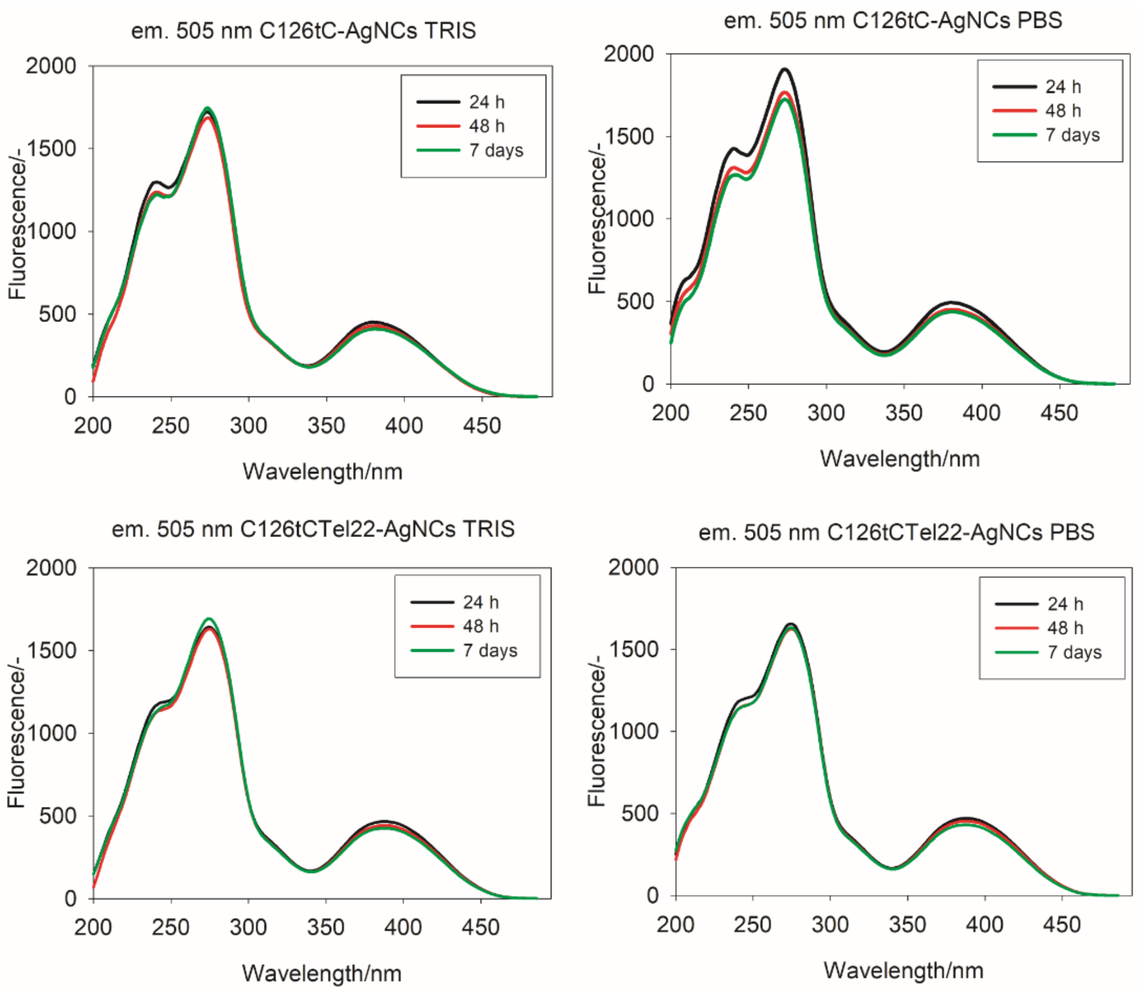

Figure S10. Fluorescence excitation spectra of C126tC-AgNCs and C126tCTel22-AgNCs at emission wavelength of 505 nm in TRIS (a, c) and PBS (b, d) buffers. Conditions: DNA (2 μM), Tris-acetate (TRIS) buffer, pH = 7.4 (0.01 M), PBS buffer, pH = 7.4 (0.01 M), and DNA:Ag^+:BH_{4^-} = 2:1:1. Ex bandwidth: 10 nm; Em bandwidth: 10 nm; response, 0.2 sec; sensitivity: low; measurement range, 280-750 nm; scan speed, 1000 nm/min.

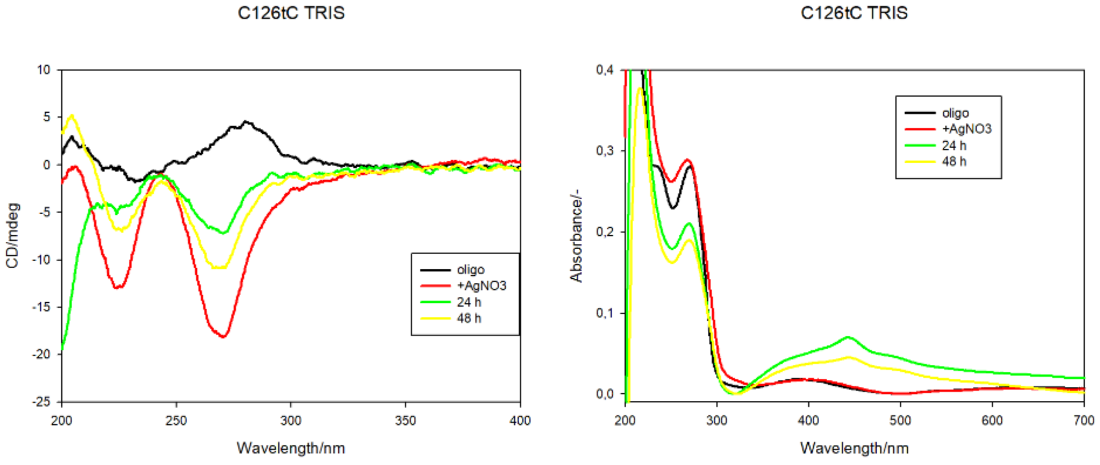

Figure S11. Circular dichroism (left) and absorbance (right) spectra of C126tC over time in TRIS buffer. Conditions: DNA (2 mM), Tris-acetate (TRIS) buffer, pH = 7.4 (0.01 M), and DNA:Ag^+:BH_{4^-} = 2:1:1

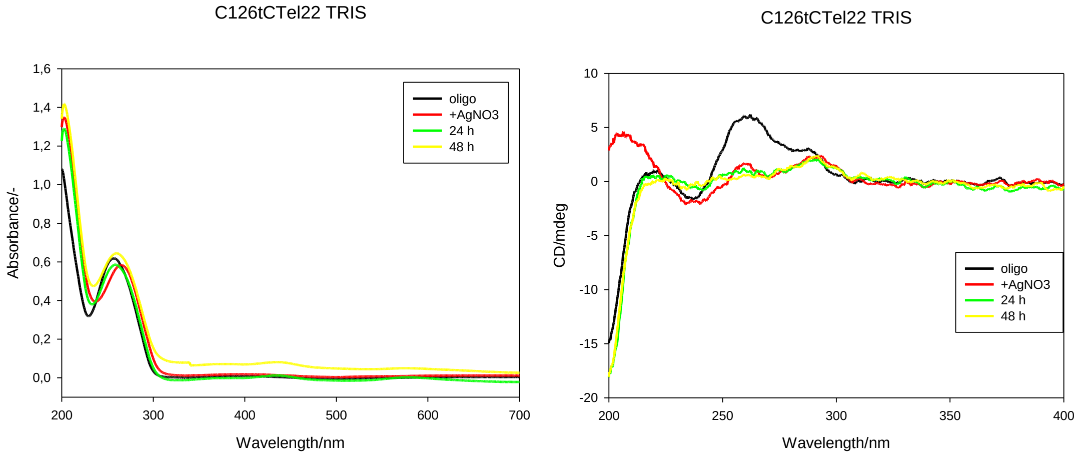

Figure S12. Absorbance (left) and circular dichroism (right) spectra of C126tCTel22-AgNCs over time in TRIS buffer. Conditions: DNA (2 mM), Tris-acetate (TRIS) buffer, pH = 7.4 (0.01 M), and DNA:Ag^+:BH_4^- = 2:1:1.

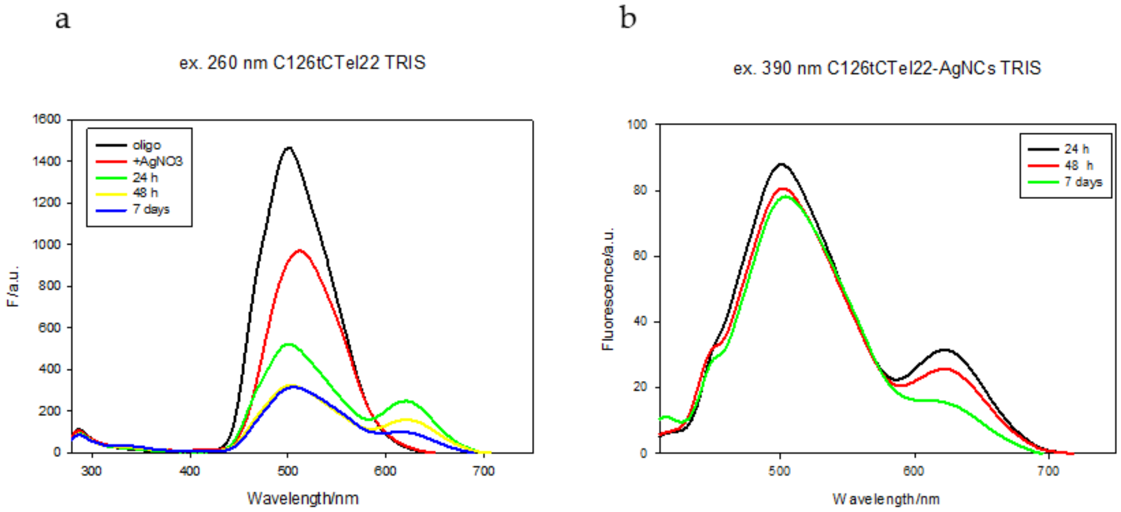

Figure S13. Changes over time of fluorescence spectra of C126tCTel22-AgNCs in TRIS excited at 260 nm (a) and 390 nm (b). Conditions: DNA (2 μM), Tris-acetate (TRIS) buffer, pH = 7.4 (0.01 M), and DNA:Ag^+:BH_4^- = 2:1:1. Ex bandwidth of 10 nm, em bandwidth of 10 nm, response of 0.2 sec, and sensitivity: medium.

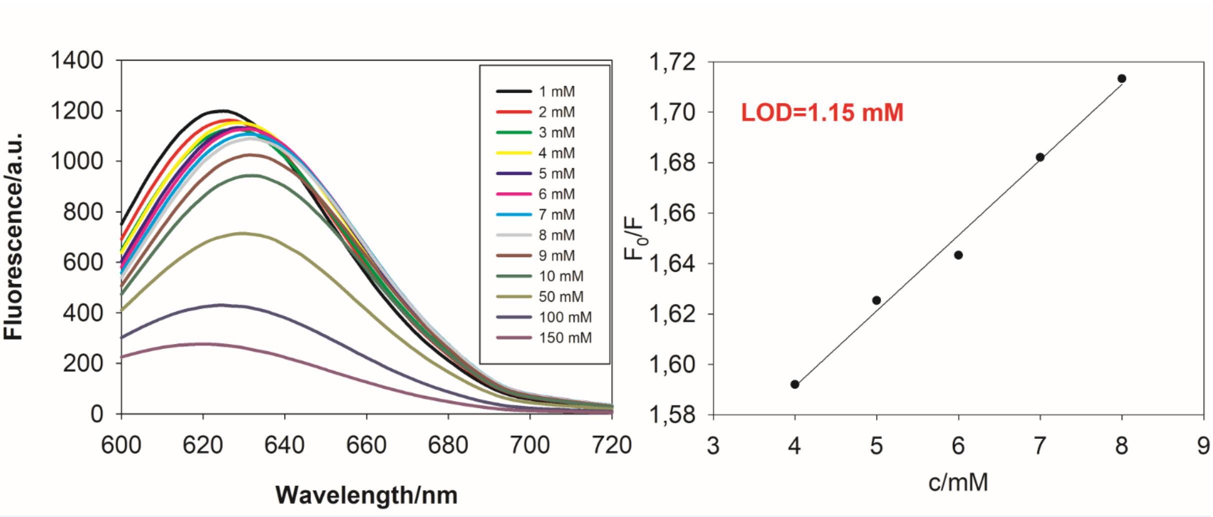

Figure S14. Fluorescence response of the C12Tel22-AgNCs in TRIS buffer at different K^+ concentrations: the emission spectra of C12Tel22-AgNCs upon adding increasing concentrations of K^+ ions (0–150 mM) (left) and corresponding Stern–Volmer plot showing K^+ quenching effect on C12Tel22-AgNC emission spectra, with λ_{max}=620 nm. Conditions: DNA (2 μM), PBS buffer, pH = 7.4 (0,01 M), DNA:Ag^+:BH_4^-=2:1:1, and K^+ 0-150 mM. Ex bandwidth of 10 nm, em bandwidth of 10 nm, response of 0.2 sec, and sensitivity: medium.

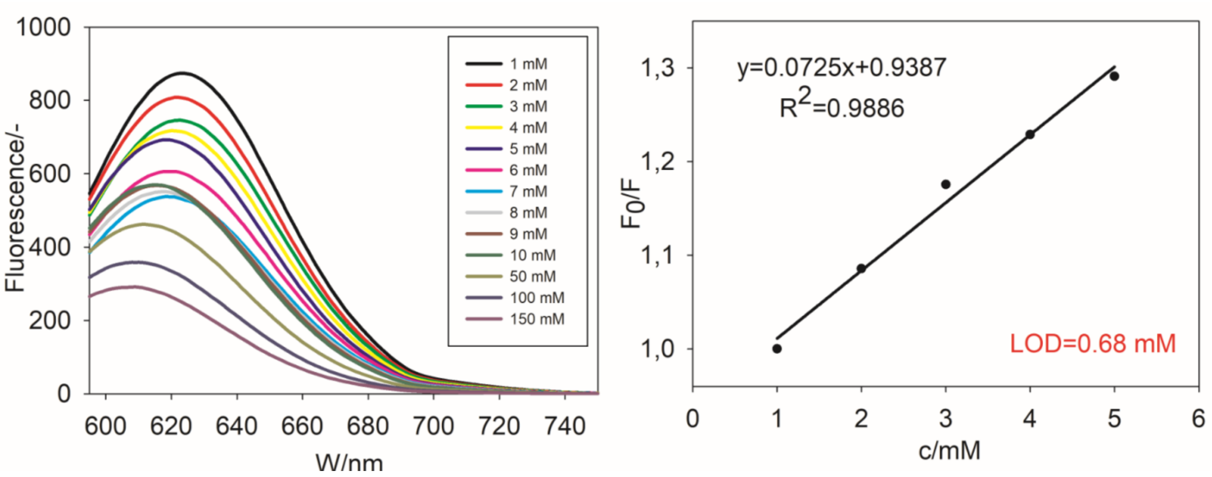

Figure S15. Fluorescence response of the C12Tel22-AgNCs in TRIS buffer containing 100 mM NaCl at different K^+ concentrations: the emission spectra of C12Tel22-AgNCs upon adding increasing concentrations of K^+ ions (0–150 mM) (left) and corresponding Stern–Volmer plot showing K^+ quenching effect on C12Tel22-AgNC emission spectra, with λ_{max}=620 nm. Conditions: DNA (2 μM), TRIS buffer, pH = 7.4 (0,01 M), DNA:Ag^+:BH_4^- = 2:1:1, and K^+ 0-150 mM. Ex bandwidth of 10 nm, em bandwidth of 10 nm, response of 0.2 sec, and sensitivity: medium.

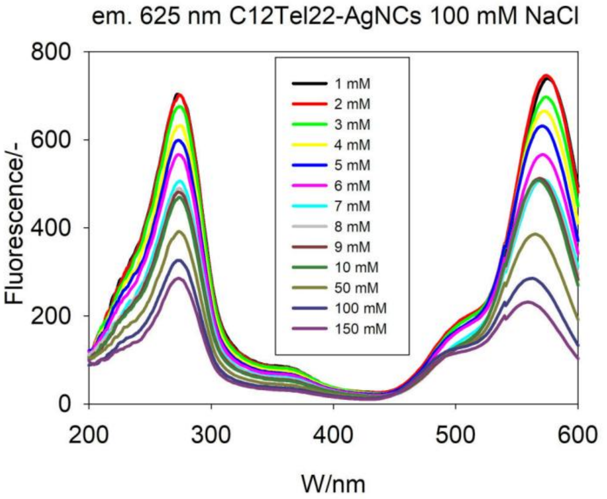

Figure S16. Fluorescence excitation spectra of the C12Tel22-AgNCs at emission wavelength of 625 nm in TRIS buffer containing 100 mM NaCl upon adding increasing concentrations of K^+ ions (0–150 mM). Conditions: DNA (2 μM), TRIS buffer, pH = 7.4 (0,01 M), DNA:Ag^+:BH_{4^-} = 2:1:1, and K^+ 0-150 mM. Ex bandwidth of 10 nm, em bandwidth of 10 nm, response of 0.2 sec, and sensitivity: medium.

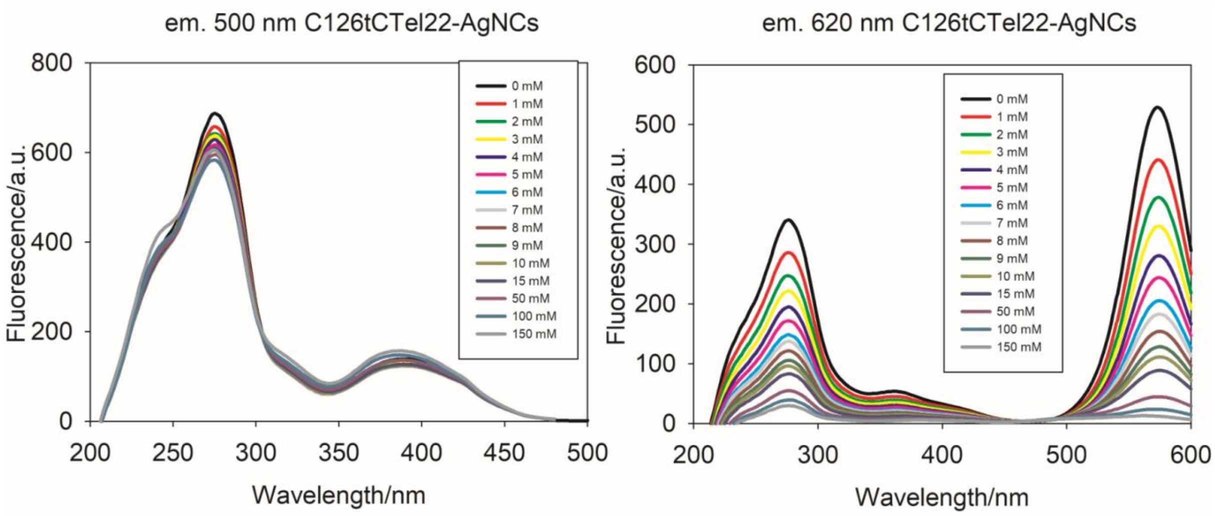

Figure S17. Fluorescence excitation spectra of the C12Tel22-AgNCs at emission wavelengths of 500 nm (left) and 620 nm (right) in TRIS buffer upon adding increasing concentrations of K^+ ions (0–150 mM). Conditions: DNA (2 μM), TRIS buffer, pH = 7.4 (0,01 M), DNA:Ag^+:BH_{4^-} = 2:1:1, and K^+ 0-150 mM. Ex bandwidth of 10 nm, em bandwidth of 10 nm, response of 0.2 sec, and sensitivity: medium.

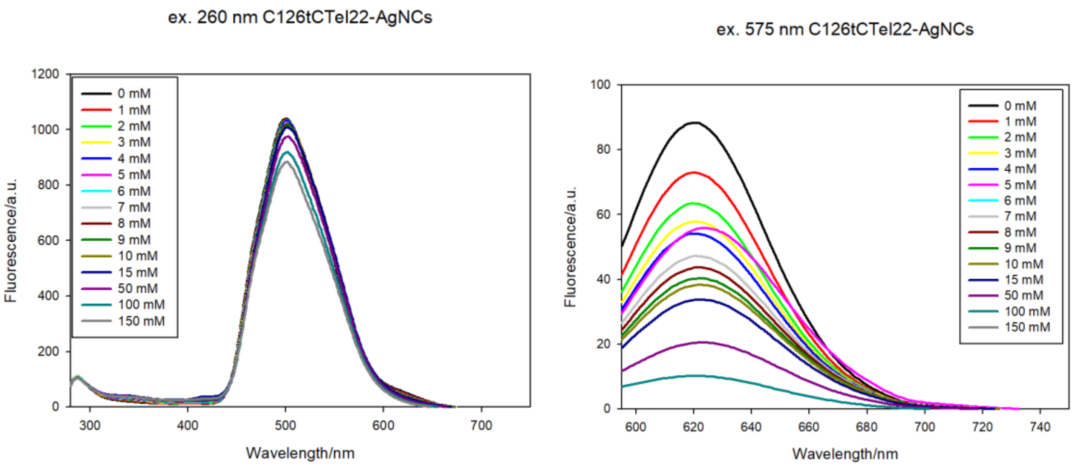

Figure S18. Fluorescence emission spectra of C126tCTel22-AgNCs during KCl titration, at excitation wavelengths of 260 nm (left) and 575 nm (right) in PBS buffer. Conditions: DNA (2 μM), PBS buffer, pH = 7.4 (0.01 M), DNA:Ag^{+}:BH_{4^-} = 2:1:1, and K^{+} 0-150 mM. Ex bandwidth of 10 nm, em bandwidth of 10 nm, response of 0.2 sec, and sensitivity: medium.

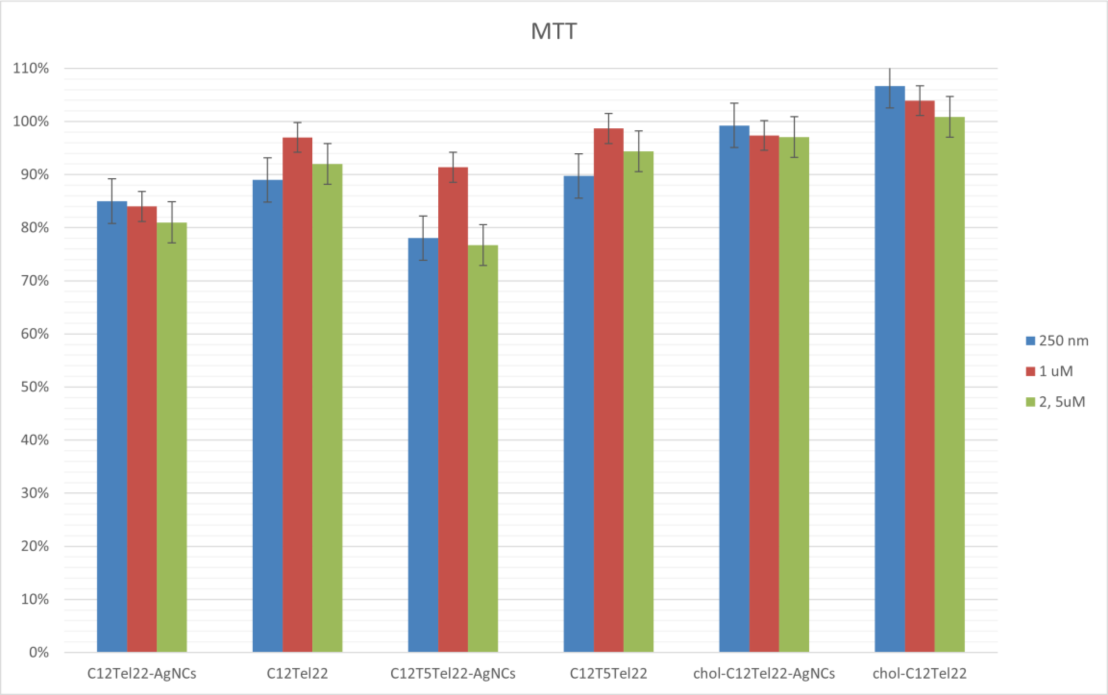

Figure S19. Effect of the tested oligonucleotides and silver nanoclusters (concentration range 0.25–2.5 μM) on the viability of Hela cells after 24 h incubations. Data presented as the mean ± SD.

Table S1. Changes in the Cell Index (CI) from 22.5 h to 96 h in HeLa cells treated with G-rich oligonucleotides and their silver nanocluster conjugates.

**Condition**	**CI @ (47 h)**	**%**	**CI @ (74 h)**	**%**	**CI @ (96 h)**	**%**
**Control**	2.8912	100%	4.1549	100%	3.9325	100%
NC-AS1411-T5 250 nM	2.2649	67%	2.9767	63%	2.8175	62%
NC-AS1411-T5 500 nM	1.5651	30%	2.4453	46%	2.6842	57%
C12Tel22 250 nM	2.9903	105%	4.1093	99%	3.6663	91%
C12Tel22 500 nM	2.7291	91%	3.9017	92%	3.4548	84%
C12Tel22-AgNCs 250 nM	2.4235	75%	2.9364	61%	2.799	61%
C12Tel22-AgNCs 500 nM	1.6823	36%	2.3087	41%	2.2129	41%
ch-C12Tel22 250 nM	2.9663	104%	4.2874	104%	3.8599	98%
ch-C12Tel22 500 nM	2.7433	92%	4.1751	101%	3.7631	94%
ch-C12Tel22-AgNCs 250 nM	2.4984	79%	3.289	73%	3.0756	71%
ch-C12Tel22-AgNCs 500 nM	2.1274	60%	3.1499	68%	3.1406	73%
